# Effects of physiological changes and social life events on adrenal glucocorticoid activity in female zoo-housed Asian elephants (*Elephas maximus*)

**DOI:** 10.1371/journal.pone.0241910

**Published:** 2020-11-06

**Authors:** Sharon S. Glaeser, Katie L. Edwards, Nadja Wielebnowski, Janine L. Brown

**Affiliations:** 1 Oregon Zoo, Portland, Oregon, United States of America; 2 Center for Species Survival, Smithsonian Conservation Biology Institute, Smithsonian National Zoological Park, Front Royal, Virginia, United States of America; 3 North of England Zoological Society, Chester Zoo, Upton-by-Chester, United Kingdom; University of Tasmania, AUSTRALIA

## Abstract

Ensuring good health and welfare is an increasingly important consideration for conservation of endangered species and includes breeding of individuals managed under human care. Understanding how factors in the captive environment affect individual animal wellbeing can be aided by long-term monitoring of biological functioning. This study involved longitudinal assessments (4 to 28 years) of reproductive and adrenal hormones in zoo-housed female Asian elephants (*Elephas maximus*) (age range 4 to ~71 years) to elucidate patterns in adrenal glucocorticoid (GC) activity in association with reproductive and demographic factors, and examine individual response to major social changes. Concentrations of serum and urinary cortisol covaried more consistently with physiological changes (ovarian cycle phase, puberty, pregnancy, lactational anestrus, and age) than with social life events (births, deaths, and facility transfers). Cortisol fluctuated across the ovarian cycle with mean concentrations being higher in the follicular than in the luteal phase, and concentrations were highest in lactational anestrous compared to all other reproductive states. The elephants in this study exhibited substantial individuality in adrenal GC response to major social change, reinforcing the need to assess welfare on an individual basis and to consider factors influencing the impact of perceived stressors, such as social relationships, social support, temperament, and life history. Outcomes from this study deepen our understanding of Asian elephant physiology and highlight the importance of taking intrinsic patterns of hormone secretion into account when evaluating the impact of external factors. Finally, a better understanding of the impact of social change and resiliency in response to real and perceived stressors allows us to improve social management to enhance welfare in both captive settings and free-ranging environments.

## Introduction

Welfare of elephants, whether free-ranging or managed to varying degrees under human care, has become an increasingly important consideration for conservation and breeding efforts worldwide. From a conservation perspective, individuals with good health and welfare generally experience increased survivorship and reproduction and thus are better able to contribute to population sustainability. To assess welfare, it is important to measure indicators of physical, physiological and psychological states at both the individual and population level. Long-term monitoring helps establish baseline values for individuals, populations, and species, and allows for detection of patterns that are important to consider when evaluating responses to external stimuli. Hormone monitoring in particular can be used to assess physiological health, and is increasingly used to enhance reproduction and welfare of *ex-situ* and *in-situ* populations of various taxa [1]. Through longitudinal measures of hormones in elephants in western zoos, we have characterized basic endocrine function and determined how environmental factors (e.g., social structures, life events, climate, feeding strategy) affect gonadal and adrenal function [2, 3]. We have used this information to improve captive management through better timing of breeding (both natural and assisted), providing more social opportunities, and improving exhibit design and husbandry to enhance elephant welfare.

Indicators of stress are commonly measured as welfare outcomes, with the most frequently used indicator being glucocorticoids (GCs) secreted from the adrenal cortex in response to a variety of stimuli [4–8] both positive (e.g., birth, excitement, mating) or negative (e.g., fear, anxiety, pain) [5, 9–11]. The primary role of GCs at basal levels is energy regulation, but higher concentrations facilitate physiological changes associated with the stress response [12], which is an adaptive reaction to real or perceived stressors resulting in a suite of behavioral, physiological and neuroendocrine changes to help an organism cope and re-establish homeostasis [4, 13, 14]. Short-term stressors (e.g., fighting, hunting, predator avoidance) cause an acute response; however, prolonged exposure to physical or psychological stressors (e.g., lack of nutrition, social aggression, poor husbandry) can result in chronic activation of the hypothalamic-pituitary-adrenal (HPA) axis with high concentrations of GCs that have deleterious effects on health and welfare including immunosuppression, decreased wound healing, increased susceptibility to disease, and poor reproduction [13, 15]. In elephants, GCs have been found to increase in response to adverse environmental conditions and physiological stress, e.g., with reduced food and water availability [16, 17] and injury [18] in free-ranging African elephants; and low winter temperatures [19] and physical exertion [20] in captive Asian elephants.

Life events such as births, deaths, and facility transfers disrupt stable social groups by adding or removing individuals, and can influence physiology and behavior in elephants. Birth is generally considered a positive stimulus. Group dynamics and activities may change with focus on a calf [21], and non-maternal female elephants often participate in allomothering [22]. By contrast, loss of individuals and social bonds is generally considered a negative stimulus. Wild elephants have been observed showing empathetic behaviors towards dying and deceased conspecifics [23, 24], suggesting that death of conspecifics, in addition to disrupting social groups, can be emotionally distressing for individual elephants at least in the short-term. Similarly, disruption in group composition from poaching in wild female African elephants negatively affected reproduction and was associated with increased GCs [25]. Elephants have also shown GC increases with transportation and relocation [26–29], which can result in dissolution of social bonds or require the development of new relationships. For example, captive female elephants exhibited increased GCs and behavioral changes during the process of social introduction [30, 31]. However, it is important to note that increases in GCs in elephants occur under normal physiological conditions; e.g., diurnally in a circadian rhythm (24 hour sleep-wake cycle) with higher concentrations in the morning [32–35], seasonally, albeit not consistently in temperature-controlled captive environments [32, 36], across the estrous cycle in females [37] and during musth in males [38, 39], and in association with stage of gestation and parturition [17, 40, 41]. Increases in GCs can also indicate arousal [5]. Males in a multitude of species have shown increased GCs during rut and in response to mating stimuli [42–44] and acute exercise [45, 46].

This study involved longitudinal assessments (4 to 28 years) of reproductive and adrenal hormones in several female Asian elephants housed at two North American zoos with a longstanding history of hormone monitoring. This extensive data set allowed us to examine patterns in adrenal GC activity and differences in individual response to a number of social changes. We examined adrenal variation with reproductive state and across the estrous cycle in elephants of varying ages and parity, as well as in response to major life events such as births, deaths, and facility transfers in and out of herdmates. Outcomes from this study can help us gain a better understanding of underlying conditions affecting patterns in adrenal GC activity, and how social change may affect the welfare of Asian elephants.

## Materials and methods

### Animals and sample collection

Female Asian elephants (n = 11) were housed at two AZA accredited-zoos: Oregon Zoo (OZ, n = 3, *Elephas maximus indicus*, n = 1 *E*. *m*. *borneensis*) and the Smithsonian National Zoological Park (NZP, n = 5 *E*. *m*. *maximus*, n = 2 *E*. *m*. *indicus*). Elephants were both wild-born (n = 8) and zoo-born (n = 3); parous (n = 1), multiparous (n = 4) and nulliparous (n = 6); with an age range of 4 to ~71 years encompassing puberty through reproductive senescence (Table 1). Two females at NZP were periodically treated with a gonadotrophin releasing hormone (GnRH) vaccine to shut down ovarian steroidogenic activity to resolve uterine pathologies. Details on the successful treatment of one female with Repro-BLOC^®^ is provided by Boedeker et al. [47]; the other female also showed resolution of uterine cysts during treatment for ~1 year with Improvest (unpublished). This study was approved by the Welfare and Research Committees at the Oregon Zoo (OZ), and by the Institution Animal Care and Use Committees at the Smithsonian National Zoological Park (NZP).

**Table 1:** Female elephants included in this study. Housing facility, individual, origin, age range during study, parity, whether the individual exhibited normal ovarian cyclicity during the study, number of samples analyzed, and statistical analyses performed for each individual.

Facility	Animal ID	Origin	Age range (years)	Parity	Exhibited Normal Cycling	Sample Number	Effects analyzed using GLMMs
OZ^a^	F1OZ	Zoo-born	4–14	Parous^c^	Yes	338	RS, OC, Age, D1, T1, T2
OZ	F2OZ	Wild	~9–15	Nulliparous	Yes	229	RS, OC, Age, D1, T2
OZ	F3OZ	Zoo-born	11–26	Nulliparous	Yes	348	OC, Age, D1, D2, D3, D4, T1, T2, T5
OZ	F4OZ	Wild	~39–51	Multiparous	Yes	488	OC, Age, D2, D3, D4, T1, T2, T5, DS1
NZP^b^	F5NZ	Wild	~15–43	Multiparous	Yes^d^	1230	RS, OC, Age, D5, D6, T3, T4, T6, TS1
NZP	F6NZ	Wild	~28–41	Nulliparous	Yes	576	OC, Age, B1, B2, D5, DS2
NZP	F7NZ	Wild	~46–71	Nulliparous	Yes^d^	1121	RS, OC, Age, B2, D5, D6, T3, T4, T6
NZP	F8NZ	Zoo-born	24–28	Multiparous	Yes	166	OC, T6, TS3
NZP	F9NZ	Wild	~39–43	Multiparous	Yes	174	RS, OC, T6, TS2
NZP	F10NZ	Wild	~38–42	Nulliparous	No	233	not modelled
NZP	F11NZ	Wild	~38–43	Nulliparous	No	242	not modelled

^a^OZ = Oregon Zoo

^b^NZP = National Zoological Park

^c^Became multiparous after data collection.

^d^Not cycling during treatment with a GnRH vaccine.

RS = Reproductive state

OC = Ovarian cycle phase (see Table 2)

Age = Age analysis (see Table 3)

B# = Birth to herdmate (see Table 4)

D# = Death of herdmate (see Table 5)

T# = Transfer of herdmate (see Table 6)

TS# = Transfer of self (see Table 7)

DS# = Health decline leading to euthanasia (see Table 8)

During the study period, females at OZ were housed as a single herd or as two separate herds comprising two to four individuals each. Two to four adult bulls were present at any given time and were housed separately from each other and from females, except for breeding or for male/female socialization. Females at NZP were housed as a single herd of three initially, and then as two separate herds comprising two to five individuals after the arrival of three new elephants. One juvenile male was gradually housed separately starting around the age of 8, but occasionally was put with females for socialization until he was transferred out at age 14. An adult bull (36 years of age) was integrated into the herd at NZP in the last year of the study.

Most elephants (all for OZ; 6 of 7 for NZP) allowed blood collection (without sedation) as part of their normal management routine. Blood was collected (3–9 ml) into red top serum separator tubes from an ear or leg vein by elephant care staff. Blood was maintained at ~4°C, and then centrifuged at 1500g within a few hours of collection to separate serum. During certain periods, five elephants at NZP were not reliable for blood collection, so urine was collected by midstream catch in a cup or tube, or off the enclosure floor, and then centrifuged at 500g to remove debris within ~ 2 hours of collection. Serum and urine samples were stored at -20°C or colder until analysis. Samples were collected weekly, and in the morning to control for diurnal patterns of cortisol secretion [32].

### Immunoassays

Approximately weekly samples were analyzed routinely for progestagens and cortisol to assess reproductive and adrenal GC activity throughout the study period. Data from 1995 to 2009 was used for OZ females, and data from 1991 to 2019 for NZP females.

Cortisol concentrations in serum samples collected through 2014 were measured using a solid-phase cortisol ^125^I radioimmunoassay (RIA) (Siemens Healthcare Diagnostics Inc., Terrytown, NY, USA) following the methods of Brown *et al*. [38]. This assay was discontinued at the end of the 2014, after which serum cortisol was analyzed using a solid-phase cortisol ^125^I RIA (CortiCote, MP Biomedicals, Santa Ana, CA; catalog # 06B256440) with modifications described in Edwards *et al*. [48]. Serum cortisol measured with the MP Biomedicals RIA was not included in analysis because the females at NZP had become less reliable for weekly blood collection and urinary cortisol was measured starting in 2015.

Urinary cortisol concentrations were quantified by a double-antibody enzymeimmunoassay (EIA) adapted from Brown *et al*. [32] and modified by Edwards *et al*. [48]. It has been previously demonstrated in elephants that circulating cortisol is excreted in the urine in its native form as free cortisol [32, 49]. The EIA utilized a polyclonal rabbit anti-cortisol antibody (R4866; C.J. Munro, University of California, Davis) and horseradish peroxidase (HRP)-conjugated cortisol label (C.J. Munro, University of California-Davis, Davis, CA). Microtiter plates were pre-coated with secondary goat-anti rabbit IgG antibody (A009, Arbor Assays, Ann Arbor, MI) described by Edwards *et al*. [48]. Cortisol standards (50 μl; 0.078–20 ng/ml), controls (50 μl), and samples (50 μl; diluted 1:20 to 1:100 in phosphate buffer [0.039M NaH_2_PO_4_, 0.061M Na_2_HPO_4_, 0.15M NaCl; pH 7.0]) were added to wells in duplicate, followed by cortisol-HRP (25 μl; 1:15 000; C. Munro, University of California, Davis, CA) to all wells. The primary anti-cortisol antibody (25 μl; R4866; 1:60 000) was added to all wells except non-specific binding (NSB) wells, followed by incubation for 1 hour at room temperature (RT). Unbound components were removed by washing five times with buffer (X007, Arbor Assays), followed immediately with addition of a chromogen solution containing TMB (100 μl, X019, Arbor Assays) to each well. After incubation for 5 min at RT, the reaction was halted by adding stop solution (50 μl; X020 Arbor Assays), and optical densities were determined at 450 nm with a reference of 630 nm. Steroid cross-reactivities of the R4866 antibody were previously reported in Young *et al*. [50]. Cross reactivities for the Healthcare Diagnostics cortisol RIA antibody were as follows: cortisol 100%, prednisolone 76%, methylprednisolone 12%, 11-deoxycortisol 11.4%, prednisone 2.3%, betamethasone 1.6%, cortisone 0.98%, and corticosterone 0.94%. Cross reactivities for the MP Biomedicals cortisol RIA antibody are as follows: cortisol 100.0%, prednisolone 94.1%, 11-deoxycortisol 2.2%, prednisone 1.2%, corticosterone 1.2%, cortisone 0.8%, dexamethasone 0.8%, 17-hydroxyprogesterone <0.05%, and metyrapone <0.01%.

Progestagens in serum samples collected through 2014 were measured using a solid-phase ^125^I progesterone RIA (Siemens Healthcare Diagnostics Inc.) validated for elephants [2, 41, 51]. After this assay was discontinued, serum samples from 2015 to 2019 and all urine samples were analyzed using a double-antibody progesterone EIA modified from the single-antibody assay of Brown *et al*. [52]. Anti-mouse antibody (A008, Arbor Assays, Ann Arbor, MI) in coating buffer (X108, Arbor Assays) (150 μl at 10 μg/ml) was added to 96-well microtiter plates (Costar, Corning Life Sciences, Tewkesbury, MA) followed by incubation at RT for 15–24 hours. Unbound antibody was then washed from wells with buffer (X007, Arbor Assays). Blocking solution (250 μl; X109, Arbor Assays) was added to each well and incubated for 4 to 24 hours at RT. Blocking solution was then removed and plates were dried at RT in a desiccator cabinet, packaged in vacuum-sealed bags and stored at 4°C until use. Progesterone standards (50 μl; 0.016–4.0 ng/ml; P0130; Sigma Chemical Co.), controls (50 μl), and samples (50 μl; neat [serum]; diluted 1:20 to 1:100 [urine] in phosphate buffer [0.039M NaH_2_PO_4_, 0.061M Na_2_HPO_4_, 0.15M NaCl; pH 7.0]) were added to wells in duplicate, followed by progesterone-HRP (25 μl; 1:90,000; C. Munro, University of California, Davis, CA) to all wells. The monoclonal anti-progesterone antibody (25 μl; CL425 1:50,000) was added to all wells except NSB wells, followed by incubation for 2 hours at RT. Unbound components were removed by washing five times with buffer (X007, Arbor Assays), followed immediately by adding a chromogen solution containing TMB (100 μl, X019, Arbor Assays) to each well. After incubation for 30 min at RT, the reaction was halted by adding stop solution (50 μl; X020 Arbor Assays), and optical densities were determined at 450 nm with a reference of 630 nm. Steroid cross-reactivities of the CL425 antibody were previously reported in Rolland *et al*. [53]. Cross reactivities for the Healthcare Diagnostics progesterone RIA were as follows: progesterone 100%, 5α-pregnan-3,20-dione 9%, 17α-hydroxyprogesterone 3.4%, 5β-pregnan-3,20-dione 3.2%, 11-deoxycorticosterone 2.2%, corticosterone 0.9%, medroxyprogesterone 0.3%, 20α-dihydroprogesterone 0.2%, pregnenolone 0.1%, and testosterone 0.1%. Both progesterone antibodies cross-react with reduced pregnanes present in elephant serum [2] herein referred to as ‘progestagens.’

Cortisol assay sensitivities were 2.5 ng/ml for the RIA and 0.08 ng/ml for the EIA. Progesterone assay sensitivities were 0.05 ng/ml for the RIA and 0.02 ng/ml for the EIA. All serum and urine samples were analyzed unextracted. Urinary steroids were indexed by creatinine (Cr) concentration according to Monfort *et al*. [54]. All assays had been validated for elephants by demonstrating: (1) parallelism between dilutions of pooled serum samples to the respective standard curve preparation and (2) significant (> 90%) recovery of exogenous standard hormone added to pooled samples before analysis. The inter- and intra-assay coefficients of variation (CVs) were maintained below 15% and 10%, respectively, for all assays and sample types.

### Determination of reproductive state and ovarian cycle phase

Reproductive state (prepubertal, normal cycling, pregnant, lactational anestrus, irregular cycling, contracepted, acyclic) and ovarian cycle phase (luteal, follicular) of female elephants at OZ were previously determined by Glaeser *et al*. [55], and the methods applied to analyzing reproductive status of the NZP elephants. First, baseline hormone concentrations were calculated for each individual using an established iterative process [51] conducted in R version 3.5.2 [56] with the package hormLong [57]. For each individual, all data points with values that exceeded the mean plus 1.5 standard deviations (SD) were removed and the process repeated until all values exceeding the mean + 1.5*SD had been removed. The remaining data points defined the baseline for that individual, and the baseline cut-off was the highest value that remained after this iterative process.

Ovarian cycles were determined based on progestagen patterns [55, 58, 59] as follows: (1) the onset of the luteal phase was defined as the first sample where progestogen concentration exceeded the baseline and remained elevated for at least two consecutive weeks and with a duration of at least four weeks; 2) the onset of the follicular phase was defined as the first sample where progestogen concentration fell below the baseline and remained below the baseline for at least two consecutive weeks; 3) single point fluctuations above or below baseline were considered within the same phase as the surrounding points; 4) data points on the baseline were included in the previous phase; 5) when data were not available for a given week, and that week appeared to coincide with the start or end of a luteal phase, it was added to the luteal phase. Ovarian cycle duration was calculated as the number of weeks from the first luteal phase sample through the last follicular phase sample. Cycles with durations outside ± 2*SD of the group mean were defined as outliers [55]. Acyclicity was defined as progestagen concentrations at baseline for extended periods encompassing the timeframe of multiple estrous cycles [51].

Descriptive statistics of ovarian cycle and phase durations were calculated using Excel (Microsoft® Office Excel 2016; Microsoft, Corp., Redmond, WA, USA) for each individual, for all females combined, and for all females combined but with outliers among the group removed.

### Demographics and life events

Origin of birth, birth date, housing facility, parity, and life event data of births, deaths, and facility transfers (change in physical location) were obtained from the AZA Asian Elephant Regional Studbook [60]. During this study (1995–2009 for OZ, 1991–2019 for NZP), three elephants were born, six died, and 10 were transferred in or out of the two facilities. These data constituted social life events (events involving herdmates) and events that an individual physically experiences (transfer of themselves between facilities, birth of offspring, and their own health decline leading to euthanasia).

### Data analysis

Median, range, mean, standard deviation (SD), and coefficient of variation (CV) in serum and urinary cortisol concentrations were calculated for each individual and all elephants combined, across all reproductive states, and during periods limited to normal cycling. All descriptive statistics were calculated using Excel (Microsoft® Office Excel 2016; Microsoft, Corp., Redmond, WA, USA). Coefficients of variation (CV) in cortisol concentration were calculated for reproductive states and ovarian cycle phases. Differences between CVs were determined by the Brown-Forsythe Test [61, 62], run in R version 3.6.1 [56] using the package “onewaytests” [63]. Significance was assessed at the 0.05 level for all analyses.

Hormone data were analyzed using generalized linear mixed models (GLMMs) in MLwiN version 2.02 [64] to investigate differences in mean cortisol concentrations according to reproductive state (prepubertal, normal cycling, pregnant, lactational anestrus, irregular cycling, contracepted, acyclic), ovarian cycle phase (luteal, follicular), age, group demographics, and in response to major life events (births, deaths, transfers). GLMMs allow random effects to be incorporated into the model [65, 66] to control for non-independence of data, which in this study involved repeated serum or urine samples per subject. Kolmogorov-Smirnov normality tests and examination of plots showed distributions of hormone data were non-normal, so hormone data were log10 transformed to improve the distribution for the GLMMs.

Separate models were created for each elephant to investigate the effect of physiological and social changes on an individual-level, and with all elephants combined to investigate group-level effects. GLMMs in this study were based on models by Edwards *et al*. [67] and [68]. The dependent variable (hormone data), random effects (date of sample collection), and fixed effects were incorporated into each model. Reproductive state, ovarian cycle phase, life events, demographics (parity, origin, housing facility), and 10-year age categories were fitted individually as categorical fixed effects; age was fitted as a continuous fixed effect. Only females that exhibited normal ovarian cycles at some point during the study were modelled. Reproductive state was added as an effect only in females that exhibited a change in state during the study, with normal cycling as the reference category in all models. Age was added as an effect only in females with at least 5 years of cortisol data, and cortisol changes with age were modelled across all reproductive states and also limited to periods of normal ovarian cycles to remove reproductive state as a confounding variable. Demographic effects were limited to periods of normal cycling for comparison.

Life events were classified as pre- and post-event, with an equal duration of 30 days before and 30 days after the event (total 60 days) to assess adrenal GC response in a biologically relevant timeframe found in other studies [26, 29, 69, 70]. A second model for each event categorized the post-event into 15-day time periods to measure more acute responses, and included a third 15-day period to determine if concentrations returned to pre-event levels within 45 days. Two exceptions to this timeframe were transfers in of a herdmate (120 days pre- and 120 days post-event to account for an acclimation period of quarantine, introduction, and integration), and period of decline leading to euthanasia (final 30 days of life compared to the preceding 30 days; total 60 days). The day the event occurred was included in the post-event time period. All events experienced by individuals in this study were modelled with the exception of events having insufficient hormone data, and parturition events because they were confounded with a change in reproductive state.

The models were first run to determine differences in hormone concentration across reproductive states and between cycle phases. For females in which reproductive state or cycle phase had a significant effect, these effects were taken into account by inclusion as covariates in the models for events. For females in which reproductive state or cycle phase did not have a significant effect, these effects were not included in the models for events with the assumption that variation within the timeframe of the event was unrelated to reproductive state changes.

**Fig 1:**
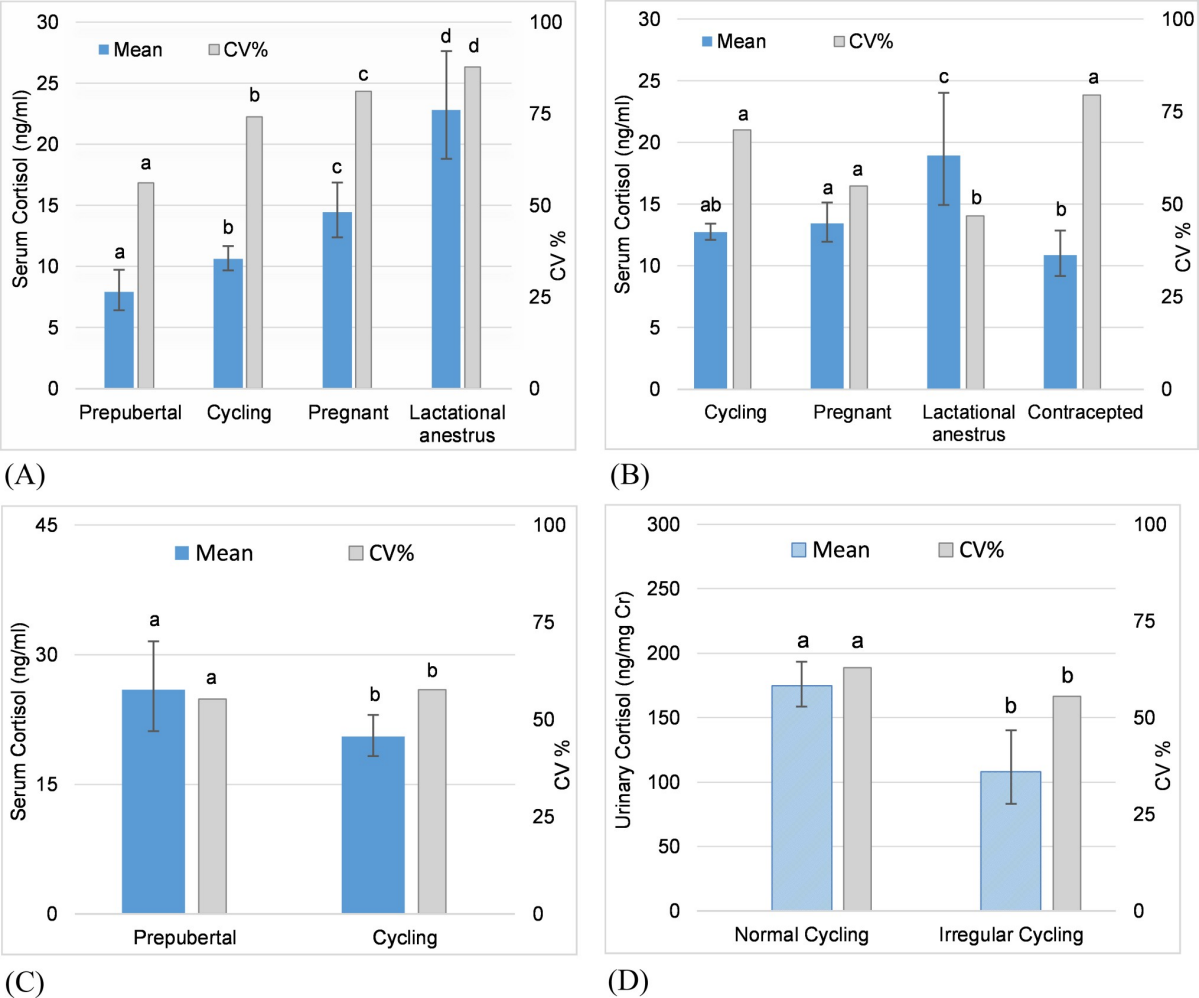
Reproductive state as a predictor of adrenal GC activity. Predictions from GLMMs for serum and urinary cortisol concentration comparing mean values across reproductive states (error bars represent the standard error of the prediction). Letters denote significant differences in mean values and in coefficients of variation (CVs) across reproductive states. (A) F1OZ: mean cortisol in prepubertal < cycling < pregnant < lactational anestrus. (B) F5NZ: mean cortisol in cycling, pregnant, contracepted < lactational anestrus. (C) F2OZ: mean cortisol in cycling < prepubertal. (D) F9NZ: mean cortisol in irregular cycling < normal cycling.

**Fig 2:**
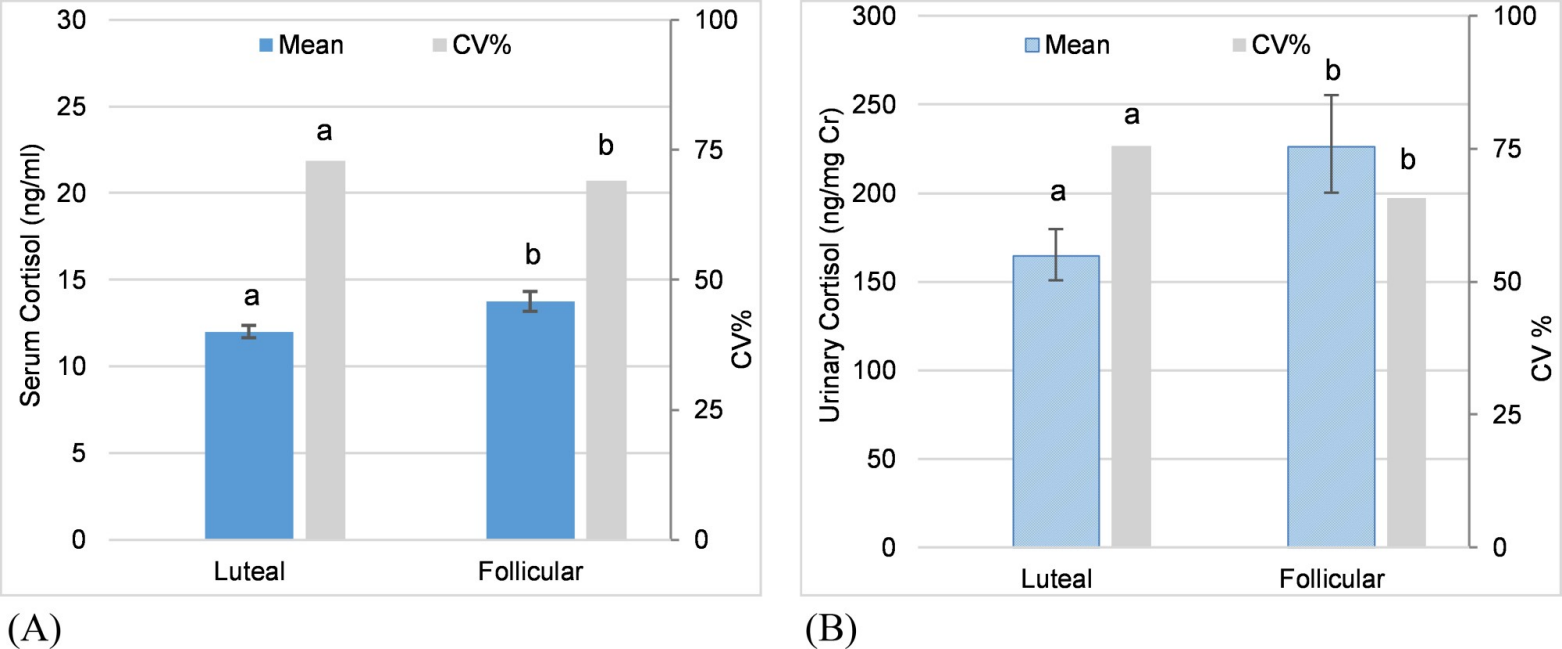
Ovarian cycle phase as a predictor of adrenal GC activity. Predictions from GLMMs for cortisol concentrations comparing mean values between the luteal phase and follicular phase of the ovarian cycle for all elephants combined (error bars represent the standard error of the prediction). Letters denote significant differences in mean values and in coefficients of variation (CVs) between cycle phases. (A) Serum cortisol in all elephants combined. (B) Urinary cortisol in all elephants combined.

**Fig 3:**
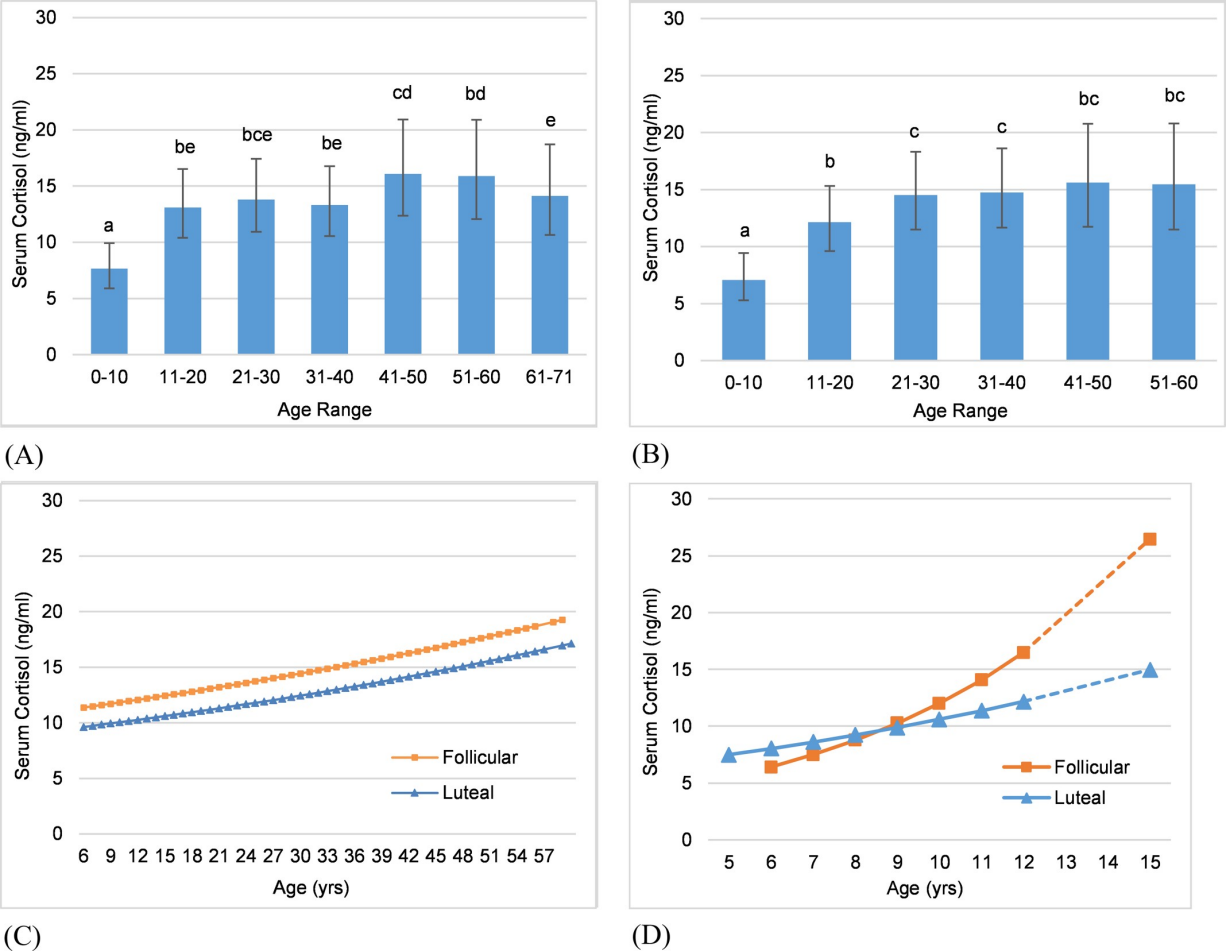
Age as a predictor of adrenal GC activity. Predictions from GLMMs for serum cortisol concentration comparing mean values across age categories (error bars represent standard error of the prediction), and interactions of age and cycle phase. Letters denote significant differences in mean values. Dashed lines in interactions indicate a reproductive state other than normal cycling. (A) All elephants: Comparing mean values across age bins and including all reproductive states. (B) All elephants: Comparing mean values across age bins and including only periods of normal cycling. (C) All elephants: Interaction of age and cycle phase showing the relationship of cortisol and cycle phase with age. (D) F1OZ: Interaction of age and cycle phase showing the relationship of cortisol and cycle phase with age.

**Fig 4:**
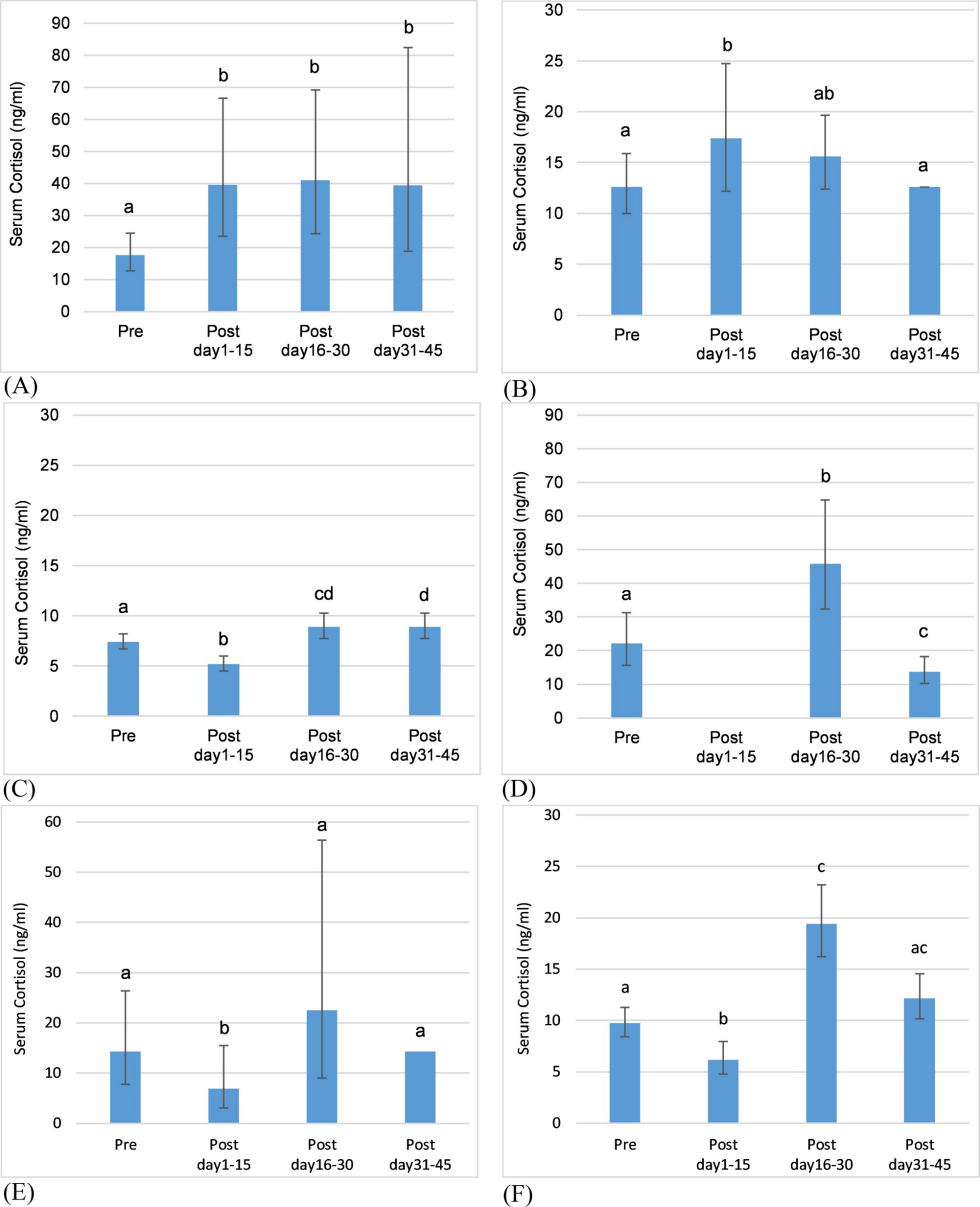
Death of a herdmate as a predictor of adrenal GC activity. Predictions from GLMMs for cortisol concentration comparing mean values in the 30 days prior to death and the 45 days post-death in 15-day time blocks (error bars represent standard error of the prediction). Letters denote significant differences in mean values across time periods. (A) Death 1, F2OZ response: Post > Pre in 15-day time periods post death; values remained above pre-event concentrations for at least 45 days. (B) Death 1, F3OZ response: Post > Pre in first 15 days post death; values returned to pre-event concentrations after 15 days. (C) Death 2, F3OZ response: values decreased then increased to above pre-event concentrations for at least 30 days. (D) Death 4, F4OZ response: values increased then decreased to below pre-event concentrations. (E) Death 5, F5NZ response: Post < Pre in first 15-days post death; values returned to pre-event concentrations after 15 days. (F) Death 5, F6NZ response: values decreased then increased above pre-event concentrations then returned to pre-event concentrations after 30 days.

**Fig 5:**
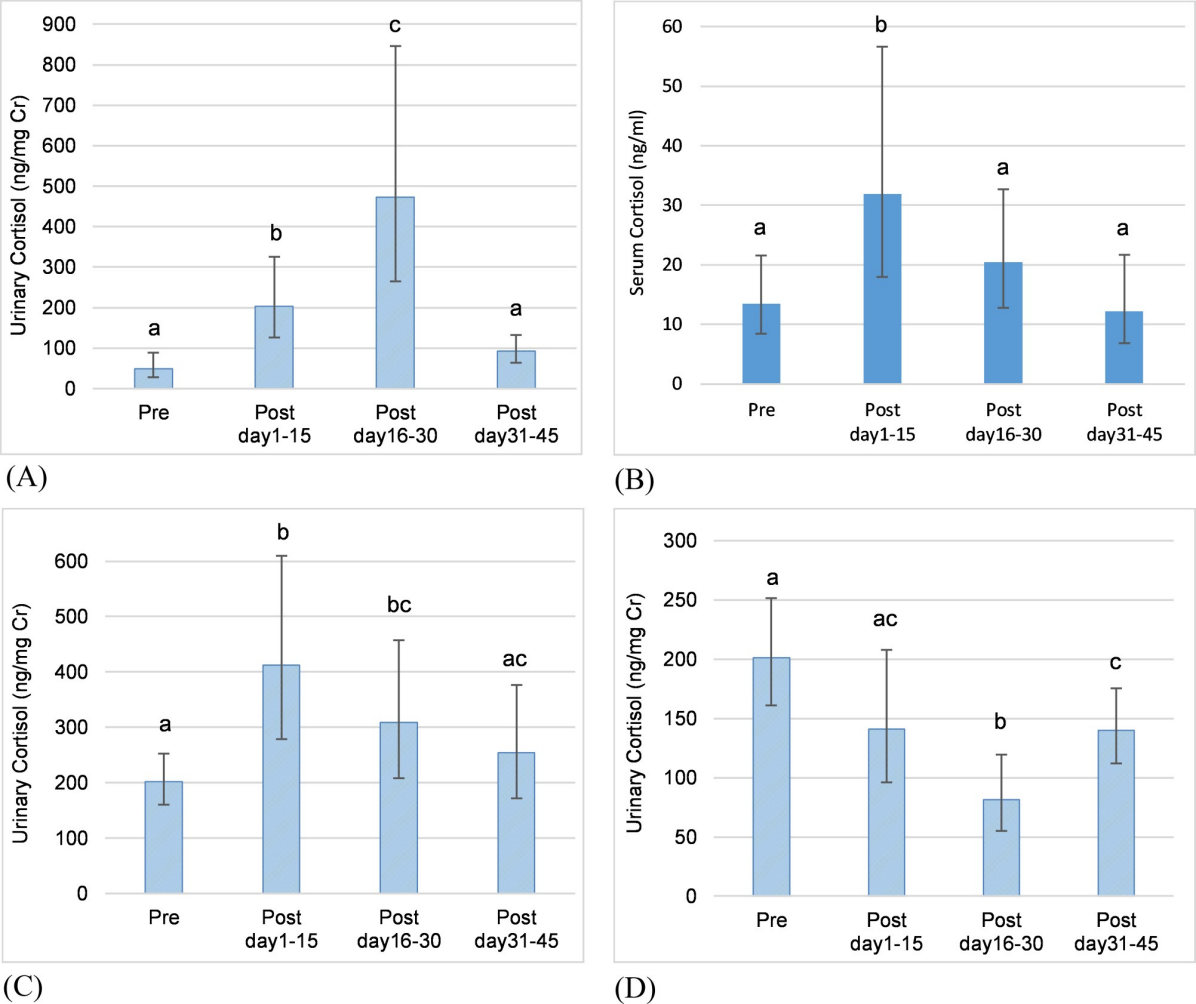
Facility transfer as a predictor of adrenal GC activity. Predictions from GLMMs for cortisol concentration comparing mean values in the 30 days prior to transfer and the 45 days post-transfer in 15-day time blocks (error bars represent standard error of the prediction). Letters denote significant differences in mean values across time periods. (A) Transfer 6 of herdmate, F9NZ response: Post > Pre in the first 15 days and increased again in the next 15 days; values returned to pre-event concentrations after 30 days. (B) F5NZ transfer of self: Post > Pre in the first 15 days after the transfer; values returned to pre-event concentrations after 15 days. (C) F8NZ transfer of self: Post > Pre in the first 30 days after transfer; values returned to pre-event concentrations after 30 days. (D) F9NZ transfer of self: No significant difference in the first 15 days, then Post < Pre for at least the next 30 days.

A Normal error structure was used for all models. The significance of each fixed effect compared to the reference category was determined using the Wald statistic and chi-squared (χ^2^) distribution, with alpha set to 0.05; and significance of pair-wise comparisons was determined using χ^2^ with alpha set to 0.05. A post-hoc power analysis was conducted on all models with a significant or close to significant effect to determine the minimum number of samples required to test the effect of cycle phase and life events on hormone concentration while keeping the power above the 80% threshold. Predicted means and standard errors (SE) of log10 cortisol and log10 GCM concentrations were back transformed to generate charts (Figs 1–5).

## Results

Estrous cycle characteristics from female Asian elephants housed at OZ are described in Glaeser *et al*. [55]. An additional 188 estrous cycles were analyzed from five female Asian elephants at NZP (JLB, SG, unpublished). With all female elephants combined, an age range of 6 to 71 years, and a larger number of estrous cycles (n = 367), durations were found to have a greater range than previously reported [55, 71]. Estrous cycle duration ranged from 9 to 19 weeks (mean 14.5, SD 2.2 weeks), luteal phase from 4 to 17 weeks (mean 9.4, SD 2.0 weeks), and follicular phase from 2 to 15 weeks (mean 5.1, SD 2.0 weeks), with outliers for cycle duration among the group (OZ and NZP combined) removed. Each elephant’s estrous cycle characteristics remained generally consistent over time (JLB, SG, unpublished). The percentage of outlier cycles was low (2.7%), with five individuals exhibiting a total of 10 outlier cycles among the OZ and NZP elephants combined, and only five outliers associated with life events. F4OZ exhibited outliers after transfer out of a herdmate and the last cycle prior to euthanasia [55]. F6NZ and F7NZ exhibited outliers after birth to a herdmate, and F9NZ after a male subadult was transferred out. Variability in estrous cycle duration was considerably less than the variability in luteal and follicular durations for all females combined (estrous CV = 15%, luteal CV = 21%, follicular CV = 38%).

### Long-term cortisol concentrations

Cortisol concentrations were highly variable among females (see S1–S4 Tables). For all elephants combined (n = 11) and across all reproductive states, serum cortisol concentrations (N = 3840 samples, n = 7 elephants) had a mean (SD) of 16.07 (11.90) ng/ml with a median (range) of 12.07 (2.50–96.00) ng/ml. For elephants or time periods when only urine was available, urinary cortisol concentrations (N = 1305 samples, n = 6 elephants) had a mean (SD) of 304.38 (299.85) ng/mg Cr with a median (range) of 223.08 (0.08–3830.12) ng/mg Cr. Variability in cortisol across individuals, as determined by the CV, ranged from 46.8% to 84.3% in serum; and 39.4% to 91.3% in urine.

For all females that exhibited normal ovarian cycling combined (n = 9), serum cortisol concentrations (N = 2989, n = 7) resulted in the following means (SD) and medians (range): mean 15.64 (11.32) ng/ml and median 12.37 (2.50–96.00) ng/ml across the cycle; mean 14.94 (10.88) ng/ml and median 11.84 (2.50–79.30) ng/ml during the luteal phase; and mean 16.88 (11.66) ng/ml and median 13.70 (2.50–96.00) ng/ml during the follicular phase. Urinary cortisol concentrations (N = 504, n = 3) resulted in the following means (SD) and medians (range): mean 239.57 (179.22) ng/mg Cr and median 197.55 (0.08–1409.60) ng/mg Cr across the cycle; mean 216.22 (163.30) ng/mg Cr and median 186.26 (0.08–1409.60) ng/mg Cr during the luteal phase; and mean 276.56 (181.60) ng/mg Cr and median 242.18 (35.23–1000.00) ng/mg Cr during the follicular phase.

### Reproductive state and adrenal GC activity

Reproductive state (prepubertal, normal cycling, pregnant, lactational anestrus, irregular cycling, contracepted, acyclic) was a significant predictor of mean cortisol concentration in four of the five individuals who changed state at least once during the study, although the pattern varied among individuals. Two females experienced four reproductive states each, and three females experienced two reproductive states each.

In two females that started cycling during the study, female F2OZ (Fig 1C) had higher mean serum cortisol concentrations (GLMM coefficient = 0.130, SE = 0.044, χ^2^ = 8.699, df = 1, p = 0.003), but lower variability (see S5 Table) in the prepubertal state compared to the cycling state; whereas female F1OZ (Fig 1A) exhibited lower mean serum cortisol concentration (GLMM coefficient = -0.127, SE = 0.051, χ^2^ = 6.284, df = 1, p = 0.012) and variability (see S5 Table) in the prepubertal state.

In two females that gave birth, serum cortisol concentrations were highest during lactational anestrus. In female F1OZ (Fig 1A) there was a significant difference between all states, with mean cortisol concentrations being higher during lactational anestrus compared to pregnancy (χ^2^ = 13.24, df = 1, p < 0.001), during pregnancy compared to cycling (GLMM coefficient = 0.134, SE = 0.040, χ^2^ = 11.24, df = 1, p = 0.001), and during cycling compared to the prepubertal state (GLMM coefficient = -0.127, SE = 0.051, χ^2^ = 6.284, df = 1, p = 0.012). Variability in cortisol concentrations followed the same pattern as the mean, with the CV being highest during lactational anestrus, followed by pregnancy, then cycling, and lowest in the prepubertal state (see S5 Table). In female F5NZ (Fig 1B), mean cortisol concentrations were higher during lactational anestrus compared to pregnancy (χ^2^ = 6.411, df = 1, p = 0.011), cycling (GLMM coefficient = 0.172, SE = 0.054, χ^2^ = 10.827, df = 1, p = 0.001), and contracepted (χ^2^ = 14.043, df = 1, p = 0.002) states. There was no difference between pregnant and cycling states (p = 0.399). In contrast to F1OZ, variability in cortisol in F5NZ was lowest in lactational anestrus with no significant difference in CV across other reproductive states (see S5 Table). Female F5NZ gave birth to two calves during the study, and her mean serum cortisol concentrations and variability were lower with the second calf (male) compared to the first calf (female) during pregnancy (first calf: mean = 16.3 ng/ml, CV = 55.8%; second calf: mean = 13.5 ng/ml, CV = 51.2%) and lactational anestrus (first calf: mean = 26.1 ng/ml, CV = 49.8%; second calf: mean = 18.6 ng/ml, CV = 39.4%).

One female, F9NZ (Fig 1D), exhibited a period of irregular cycling [characterized by a either a long follicular phase (mean + 7SD) or a luteal phase with concentrations close to baseline], and mean cortisol was lower during irregular cycling compared to normal cycling (GLMM coefficient = -0.209, SE = 0.062, χ^2^ = 11.45, df = 1, p < 0.001); variability also was lower during irregular cycling (see S5 Table).

In two females that were contracepted to resolve uterine pathologies, mean cortisol concentrations were lower during the contracepted state compared to cycling, trending toward significance for serum (F5NZ: GLMM coefficient = -0.069, SE = 0.039, χ^2^ = 3.145, df = 1, p = 0.076; F7NZ: GLMM coefficient = -0.034, SE = 0.017, χ^2^ = 3.790, df = 1, p = 0.052), but not for urine (F5NZ: GLMM coefficient = -0.040, SE = 0.049, χ^2^ = 0.687, df = 1, p = 0.407). Female F5NZ resumed normal cycling approximately 1 year after the last GnRH booster; whereas female F7NZ was contracepted at age 59 and never resumed cycling again even after the GnRH antibody titers returned to baseline.

### Ovarian cycle phase and adrenal GC activity

In females that exhibited normal ovarian cycles (n = 9), cycle phase was a significant predictor of mean cortisol in all females combined and in four individuals (Table 2), with lower concentrations during the luteal compared to the follicular phase (Fig 2). There was no significant difference in mean cortisol between cycle phases in five individuals; no females showed higher cortisol during the luteal phase. Post-hoc power analysis indicated that the number of samples was greater than the minimum required to achieve 80% power in all cases except two, where the power was reduced to 75% and 70%. Variability in cortisol concentrations was higher during the luteal phase than the follicular phase for all elephants combined. Only those females with a statistical difference in mean cortisol concentrations between cycle phases also exhibited a statistical difference in variability, although the patterns varied (see S6 Table).

**Table 2:** Ovarian cycle phase as a predictor of adrenal GC activity. Individual, sample type, effect size with standard error (SE), Wald statistic, and p-value from GLMMs, and whether the mean and coefficient of variation (CV) in cortisol in the luteal phase was higher or lower than in the follicular phase. Degrees of freedom (df) was 1 in all pair-wise comparisons.

Individual(s)	Sample Type	Effect Size (SE)	Wald	P	Luteal Phase Relative to Follicular Phase	
					Mean	CV
All elephants	Serum	-0.059 (0.011)	27.559	**< 0.001**	Lower	Higher
All elephants (all parous)	Urine	-0.138 (0.033)	17.390	**< 0.001**	Lower	Higher
Multiparous females	Serum	-0.078 (0.018)	18.930	**< 0.001**	Lower	Higher
Nulliparous females	Serum	-0.041 (0.013)	9.318	**0.002**	Lower	Higher
F1OZ	Serum	-0.098 (0.040)	6.126	**0.013**	Lower^a^	Lower
F5NZ	Serum	-0.121 (0.025)	23.363	**0.001**	Lower	Higher
	Urine	-0.098 (0.037)	6.964	**0.008**	Lower^b^	Lower
F7NZ	Serum	-0.082 (0.021)	15.132	**< 0.001**	Lower	Lower
F8NZ	Urine	-0.229 (0.065)	12.438	**< 0.001**	Lower	Higher
F3OZ	Serum	-0.066 (0.034)	3.678	0.055	--	--
F2OZ	Serum	0.054 (0.055)	0.977	0.323	--	--
F4OZ	Serum	0.000 (0.031)	--	1.000	--	--
F6NZ	Serum	-0.009 (0.019)	0.212	0.645	--	--
F9NZ	Urine	-0.033 (0.048)	0.472	0.492	--	--

-- No significant difference

^a^ Power = 70%

^b^ Power = 75%

With regards to parity, cycle phase was a significant predictor of mean serum cortisol for parous and multiparous females combined (n = 5) and nulliparous females combined (n = 4), with lower concentration during the luteal phase compared to the follicular phase in both groups (Table 2), and higher variability during the luteal compared the follicular phase (Table 2). Although these patterns were the same for both groups, the interaction of parity and cycle phase was significant (GLMM coefficient = 0.039, SE = 0.015, χ^2^ = 6.84, df = 1, p = 0.009), with cycle phase having a larger effect on mean cortisol in parous and multiparous females than in nulliparous females. Furthermore, among parous and multiparous females, three females exhibited lower mean cortisol concentrations in the luteal phase and two exhibited no significant difference; among nulliparous females, one exhibited lower mean cortisol concentrations in the luteal phase and three exhibited no significant difference (Table 2).

### Demographics factors and adrenal GC activity

In normal cycling females, mean serum cortisol concentrations (N = 2989 samples, n = 7 elephants) did not differ between wild-born (n = 5) and zoo-born (n = 2) females (GLMM coefficient = -0.097, SE = 0.076, χ^2^ = 1.627, df = 1, p = 0.202), or between housing facilities (OZ: n = 4, NZP: n = 3) (GLMM coefficient = -0.045, SE = 0.075, χ^2^ = 0.362, df = 1, p = 0.547). Urinary cortisol could not be similarly compared because all females (n = 3) were at one facility and only one was zoo-born.

For females combined across all reproductive states (n = 7 elephants with at least 5 years of cortisol data), there was no overall change in mean cortisol concentration with age, although differences were observed across the 10-year age categories (Table 3). Mean cortisol was lowest in the 0–10 years age category, remained higher in the age categories spanning 11–60 years with 41–60 years being highest, and then decreased in the oldest age category (>61 years of age) (Fig 3A).

**Table 3:** Age as a predictor of adrenal GC activity. Individual, age range of analysis, age variable (age, age category, interaction of age and cycle), effect size with standard error (SE), Wald statistic, and p-value from GLMMs; and relative effect of age if significant. Age effect for individuals was modelled only during periods of normal cycling. Degrees of freedom (df) was 1 in all pair-wise comparisons. Females listed in order of age during analysis.

Individual	Age Range of Analysis	Age Variable	Effect Size (SE)	Wald	P	Age Effect
All elephants: Across all reproductive states	4–71	Age (years)	0.001 (0.001)	1.225	0.289	--
		0–10 (reference)	-	-	-	
		11–20	0.233 (0.029)	66.355	**<0.001**	Higher
		21–30	0.256 (0.034)	57.729	**<0.001**	Higher
		31–40	0.240 (0.035)	46.526	**<0.001**	Higher
		41–50	0.323 (0.054)	36.130	**<0.001**	Higher
		51–60	0.317 (0.057)	30.739	**<0.001**	Higher
		61–71	0.266 (0.059)	20.506	**<0.001**	Higher
All elephants: During normal cycling only	6–59	Age (years)	0.005 (0.001)	14.590	**<0.001**	Increasing
		0–10 (reference)	-	-	-	
		11–20	0.235 (0.041)	32.549	**<0.001**	Higher
		21–30	0.312 (0.046)	45.937	**<0.001**	Higher
		31–40	0.319 (0.048)	44.704	**<0.001**	Higher
		41–50	0.344 (0.072)	22.786	**<0.001**	Higher
		51–60	0.340 (0.075)	20.780	**<0.001**	Higher
		Interaction of Age and Cycle	0.000 (0.001)	0.160	0.689	--
F1OZ	6–15	Age (years)	0.044 (0.007)	39.33	**<0.001**	Increasing
		0–10 (reference)	-	-	-	
		11–20	0.242 (0.036)	44.501	**<0.001**	Higher
		Interaction of Age and Cycle	-0.039 (0.014)	7.254	**0.007**	Significant
F2OZ	~12–15	Age (years)	0.006 (0.024)	0.055	0.816	--
		Interaction of Age and Cycle	0.027 (0.049)	0.321	0.571	--
F3OZ	11–26	Age (years)	0.019 (0.004)	20.734	**<0.001**	Increasing
		11–20 (reference)	-	-	-	
		21–30	0.154 (0.034)	20.572	**<0.001**	Higher
		Interaction of Age and Cycle	0.005 (0.008)	0.418	0.518	--
F4OZ	~39–51	Age (years)	0.017 (0.004)	15.120	**<0.001**	Increasing
		31–40 (reference)	-	-	-	
		41–50*	0.047 (0.074)	0.406	0.524	--
		51–60*	0.219 (0.093)	5.842	**0.019**	Higher
		Interaction of Age and Cycle	0.012 (0.009)	1.647	0.199	--
F5NZ (serum)	~15–37	Age (years)	0.000 (0.002)	0.017	0.896	--
		11–20 (reference)	-	-	-	
		21–30	0.035 (0.035)	0.983	0.321	--
		31–40	0.017 (0.037)	0.213	0.644	--
		Interaction of Age and Cycle	-0.002 (0.004)	0.221	0.638	--
F5NZ (urine)	~39–43	Age (years)	0.124 (0.014)	82.255	**<0.001**	Increasing
		Interaction of Age and Cycle	-0.040 (0.029)	2.007	0.157	--
F6NZ	~28–41	Age (years)	0.010 (0.003)	15.888	**<0.001**	Increasing
		21–30 (reference)	-	-	-	
		31–40	0.100 (0.024)	17.393	**<0.001**	Higher
		Interaction of Age and Cycle	0.003 (0.005)	0.285	0.593	--
F7NZ (serum)	~46–59	Age (years)	-0.080 (0.003)	7.159	**0.007**	Decreasing
		41–50 (reference)	-	-	-	
		51–60	-0.041 (0.020)	4.260	**0.039**	Lower
		Interaction of Age and Cycle	-0.008 (0.006)	1.760	0.185	--
F7NZ (urine)**	~67–71	Age (years)	0.103 (0.011)	95.443	**<0.001**	Increasing

-- No significant difference

* Significant difference between time categories

** Reproductive state is acyclic

For females combined and limited to the state of normal cycling (n = 7), mean cortisol concentrations increased with age overall, and also across age categories (Table 3). Concentrations were lowest in the 0-10-year age category, higher in the 11–20 and 21–30 categories, then showed no significant increases or decreases in mean concentrations after the age of 30 years (Fig 3B). The interaction of age and cycle phase was not significant at a group level (Table 3), indicating that the relationship between cortisol and cycle phase did not change with age, but rather basal concentrations increased in both the follicular and luteal phases over time (Fig 3C). However, in female F1OZ (Fig 3D) who transitioned from pre-puberty to cycling during the study, there was a significant interaction between age and cycle phase, with a switch in the relationship between cortisol and the cycle phases (i.e., a crossover interaction) approximately 2 years after her first estrous cycle. Mean cortisol concentrations were initially lower in the follicular phase; cortisol increased in both phases but the rate of increase was higher in the follicular phase.

At an individual level and limited to the state of normal cycling, age was a significant predictor of mean cortisol concentrations in all females except F2OZ, with cortisol increasing with age in five females and decreasing with age in the oldest female, F7NZ (Table 3). In the two oldest females, F4OZ showed an overall increase with age, but no increase across age categories until age 51–60 years; whereas F7NZ showed an overall decrease with age through the 51–60 age category. Two females exhibited an increase in mean cortisol only in the last years of the study. Female F5NZ showed no increase with age until the last 4 years (age 39–43) when mean urinary cortisol concentrations increased by 168%. Female F7NZ exhibited an increase in urinary cortisol concentrations only in the last 4 years (age 67–71), during which time she was acyclic. It is possible the increased adrenal GC activity in these two females was confounded by declines in health.

### Life events and adrenal GC activity

A total of 22 life events (births, deaths, transfers) occurred during the study, representing 51 events experienced by nine elephants collectively. For 40 of the 51 events, there was sufficient hormone data to analyze the adrenal GC response, taking into account covariates of reproductive state and cycle phase. Of the 40 events, 38 were related to social life (births to a herdmate, deaths of offspring or a herdmate, and facility transfers) and two euthanasias with a preceding health decline. A social life event was a significant predictor of mean cortisol concentration in 15 of 38 events (39%) for 8 of 9 females. Females with over 10 years of cortisol data (n = 6) experienced three to seven social life events, and the percentage of significant responses ranged from 0 to 50%. Overall, this group of elephants did not show chronically elevated or suppressed concentrations of GCs during the study, and significant changes in mean cortisol concentrations returned to pre-event concentrations within 45 days in all but four cases (3 deaths of herdmates, 1 transfer of self).

Tables 4–8 show the life events that were modelled for each individual, comparing mean cortisol concentrations pre- and post-event. Post-events in 15-day periods are shown only if a significant difference was detected in any single time period compared to pre-event concentrations.

**Table 4:** Birth to a herdmate as a predictor of adrenal GC activity. Fixed effects, effect size with standard error (SE), Wald statistic, and p-value from GLMMs, and whether mean cortisol concentration post-event was higher or lower than pre-event. Degrees of freedom (df) was 1 in all pair-wise comparisons.

Event	Individual Exposed to Event	Effect Size (SE)	Wald	P	Mean Post-event Relative to Pre-event
Birth1: Female calf (nzF1) to F5NZ	F6NZ	-0.229 (0.193)	1.396	0.237	--
Birth2: Male calf (nzM1) to F5NZ	F6NZ	0.020 (0.109)	0.033	0.856	--
	F7NZ	-0.190 (0.306)	0.386	0.534	--

-- No significant difference

**Table 5:** Death to a herdmate as a predictor of adrenal GC activity. Fixed effects, effect size with standard error (SE), Wald statistic, and p-value from GLMMs, and whether the mean cortisol concentration post-event is higher or lower than pre-event. Degrees of freedom (df) was 1 in all pair-wise comparisons.

Event	Individual Exposed to Event	Comparison category	Effect Size (SE)	Wald	P	Mean Post-event Relative to Pre-event
Death 1: Female (F4OZ)	F1OZ	Post	0.090 (0.100)	0.809	0.368	--
	F2OZ	Post	0.361 (0.116)	9.727	**0.002**	Higher
		Post-day1-15	0.352 (0.137)	6.630	**0.010**	Higher
		Post-day16-30	0.369 (0.137)	7.289	**0.007**	Higher
		Post-day31-45	0.351 (0.179)	3.851	**0.050**	Higher
	F3OZ	Post	0.139 (0.065)	4.615	**0.032**	Higher
		Post-day1-15	0.139 (0.059)	5.539	**0.019**	Higher
		Post-day16-30	0.093 (0.072)	1.639	0.200	--
		Post-day31-45	0.000 (0.000)	0.000	1.000	--
Death 2: Female (ozF1)	F3OZ	Post	-0.037 (0.090)	0.167	0.683	--
		Post-day1-15	-0.153 (0.039)	15.816	**<0.001**	Lower
		Post-day16-30	0.080 (0.039)	4.315	**0.038**	Higher
		Post-day31-45	0.080 (0.039)	4.315	**0.038**	Higher
	F4OZ	Post	-0.246 (0.261)	0.888	0.346	--
Death 3: Female (ozF2)	F3OZ	Post	0.265 (0.356)	0.554	0.457	--
	F4OZ	Post	-0.273 (0.207)	1.752	0.186	--
Death 4: Male (ozM1)	F1OZ	Post	-0.346 (0.119)	8.468	**0.004**	Lower*
	F3OZ	Post	0.316 (0.238)	1.756	0.185	--
	F4OZ	Post	0.315 (0.141)	4.969	**0.026**	Higher
		Post-day1-15	no data	no data	no data	--
		Post-day16-30	0.315 (0.109)	8.353	**0.004**	Higher
		Post-day31-45	-0.210 (0.099)	4.438	**0.035**	Lower
Death 5: Female calf (nzF1)	F5NZ	Post	-0.145 (0.164)	0.785	0.376	Lower
		Post-day1-15	-0.317 (0.117)	7.293	**0.007**	Lower
		Post-day16-30	0.197 (0.151)	1.701	0.192	--
		Post-day31-45	0.000 (0.000)	0.0	1.0	--
	F6NZ	Post	-0.134 (0.144)	0.862	0.353	--
		Post-day1-15	-0.197 (0.065)	9.205	**0.002**	Lower
		Post-day16-30	0.300 (0.051)	34.097	**<0.001**	Higher
		Post-day31-45	0.097 (0.051)	3.556	0.059	--
	F7NZ	Post	-0.060 (0.075)	0.636	0.425	--
Death 6: Female (F6NZ)	F5NZ	Post	0.046 (0.114)	0.163	0.686	--
	F7NZ	Post	0.041 (0.110)	0.139	0.709	--

-- No significant difference

* 60-day model (60 days pre- and post-event)

**Table 6:** Transfer of a herdmate as a predictor of adrenal GC activity. Fixed effects, effect size with standard error (SE), Wald statistic, and p-value from GLMMs, and whether the mean cortisol concentration post-event was higher or lower than pre-event. Degrees of freedom (df) was 1 in all pair-wise comparisons.

Event	Individual Exposed to Event	Comparison category	Effect Size (SE)	Wald	P	Mean Post-event Relative to Pre-event
Transfer 1: In of female (F2OZ)	F1OZ	Post	0.006 (0.132)	0.002	0.964	--
	F3OZ	Post	-0.115 (0.116)	0.988	0.320	--
	F4OZ	Post	-0.045 (0.136)	0.109	0.741	--
Transfer 2: In of male (ozM2)	F1OZ	Post	-0.049 (0.097)	0.257	0.612	--
	F2OZ	Post	0.207 (0.093)	5.011	**0.025**	Higher
	F3OZ	Post	-0.192 (0.096)	4.024	**0.045**	Lower
	F4OZ	Post	-0.208 (0.070)	8.928	**0.003**	Lower
Transfer 3: Out of female (ozF3)	F3OZ	Post	-0.062 (0.137)	0.204	0.652	--
	F4OZ	Post	0.029 (0.202)	0.201	0.654	--
Transfer 4: In of female (F10NZ)	F5NZ	Post	0.022 (0.108)	0.043	0.325	--
	F7NZ	Post	-0.079 (0.144)	0.487	0.485	--
Transfer 5: In of female group (F9NZ, F8NZ, F11NZ)	F5NZ	Post	0.161 (0.144)	1.247	0.264	--
	F7NZ	Post	0.086 (0.098)	0.768	0.380	--
Transfer 6: Out of male (nzM1)	F5NZ	Post	0.260 (0.112)	5.387	**0.020**	Higher
		Post-day1-15*	0.260 (0.104)	6.266	**0.012**	Higher
	F7NZ	Post	-0.136 (0.168)	0.651	0.420	--
	F8NZ	Post	0.212 (0.218)	0.943	0.331	--
	F9NZ	Post	0.759 (0.189)	16.165	**<0.001**	Higher
		Post-day1-15	0.612 (0.166)	13.575	**<0.001**	Higher
		Post-day16-30	0.980 (0.182)	29.038	**<0.001**	Higher
		Post-day31-45	0.269 (0.152)	3.125	0.077	--

-- No significant difference

* GLMM showed no difference between Days 16–45 and pre-event

**Table 7:** Transfer of self as a predictor of adrenal GC activity. Fixed effects, effect size with standard error (SE), Wald statistic, and p-value from GLMMs, and whether the mean cortisol concentration post-event was higher or lower than pre-event. Degrees of freedom (df) was 1 in all pair-wise comparisons.

Event	Comparison category	Effect Size (SE)	Wald	P	Mean Post-event Relative to Pre-event
Transfer of self 1: F5NZ	Post	0.257 (0.157)	2.693	0.101	--
	Post-day1-15	0.373 (0.164)	5.146	**0.023**	Higher
	Post-day16-30	0.180 (0.147)	1.492	0.222	--
	Post-day31-45	-0.044 (0.164)	0.070	0.791	--
Transfer of self 2: F8NZ	Post	0.248 (0.073	11.349	**<0.001**	Higher
	Post-day1-15	0.310 (0.071)	19.067	**<0.001**	Higher
	Post-day16-30	0.185 (0.071)	6.791	**0.009**	Higher
	Post-day31-45	0.100 (0.071)	1.984	0.159	--
Transfer of self 3: F9NZ	Post	-0.273 (0.110)	6.203	**0.013**	Lower
	Post-day1-15	-0.153 (0.099)	2.402	0.121	--
	Post-day16-30	-0.393 (0.099)	15.805	**<0.001**	Lower
	Post-day31-45	-0.157 (0.070)	5.015	**0.025**	Lower

-- No significant difference

**Table 8:** Health decline leading to euthanasia as a predictor of adrenal GC activity. Fixed effects, effect size with standard error (SE), Wald statistic, and p-value from GLMMs, and whether the mean cortisol concentration in the final 30 days before death was higher or lower than the previous 30 days. Degrees of freedom (df) is 1 in all pair-wise comparisons.

Event	Effect Size (SE)	Wald	P	Mean Final 30 Days Relative to Previous 30 Days
Health decline 1: F4OZ	-0.225 (0.184)	1.497	0.221	--
Health decline 2: F6NZ	0.355 (0.088)	16.132	**<0.001**	Higher

-- No significant difference

In cases where mean cortisol concentrations decreased in response to an event, pre-event concentrations for the individual were compared to their means across all reproductive states and during normal cycling (see S1–S4 Tables) while accounting for increases in cortisol with age. This increases the likelihood that the observed decrease following an event did not represent a return to basal concentrations, but rather a response to the event itself.

#### Births to herdmates

Of the three births that occurred, two had sufficient data to model. Birth to a herdmate was not a significant predictor of mean cortisol concentration (Table 4).

#### Deaths of herdmates

Six deaths occurred during the study, 4 at OZ (3 adult females, 1 adult male) and 2 at NZP (1 female calf, 1 adult female), representing 15 death events experienced by 7 elephants collectively; all were modelled. Death of a herdmate was a significant predictor of mean cortisol concentration in 7 (47%) of the events modelled (Table 5). The response to the same death varied among individuals (in 4 of 6 deaths), and individuals exhibited different responses to the deaths of different herdmates (in 4 of 5 individuals that experienced multiple deaths) (Table 5).

The death of F4OZ had a significant effect on 2 of 3 herdmates, with higher mean cortisol concentrations in the time periods following her death (Table 5). Female F2OZ exhibited increased cortisol for at least 45 days (Fig 4A); whereas F3OZ showed an increase only in the first 15 days (Fig 4B). The death of female ozF1 had a significant effect on one of two herdmates. Female F3OZ exhibited decreased cortisol in the first 15 days, then concentrations increased compared to pre-event concentrations for at least the next 30 days (Fig 4C). The deaths of females ozF2 and F6NZ had no significant effect on mean cortisol concentration of herdmates.

The death of female calf nzF1 (at age 1.4 years) had a significant effect on 2 of 3 females. In both individuals, mean cortisol was lower following her death (Table 4). The mother of the calf, F5NZ, showed a decrease in cortisol in the first 15 days, then returned to pre-event concentrations (Fig 4E), whereas F6NZ showed a decrease in cortisol for the first 15 days then an increase and a return to pre-event concentrations within 45 days (Fig 4F).

The death of male ozM1 had a significant effect on 2 of 3 females. F4OZ exhibited higher mean cortisol concentrations in the 30-day time period following his death (Table 5) but showed increased concentrations only in days 16–30 following his death (Fig 4D). F1OZ exhibited decreased cortisol in the 60-day time period following his death and a 71% decrease in the 30-day time period.

#### Transfers of herdmates

Ten individuals were transferred during the study, 3 at OZ (2 transfers in, 1 transfer out) and 7 at NZP (6 transfers in, 1 transfer out), with 7 transfers of single elephants and 1 group transfer. Of these 8 transfer events, 6 were modelled for transfer of herdmate, representing 17 herdmate transfer events experienced by 8 elephants collectively. The transfer in of F5NZ could not be modelled for herdmates because there was not sufficient data. The transfer in of an adult male to NZP in the last year of the study was not modelled because only 2 of 4 females had sufficient cortisol data, and both of those had confounding health declines. Transfer of a herdmate was a significant predictor of mean cortisol concentration in 5 (29%) of the 17 events (Table 6), all of which were in response to transfers of 2 males, although responses differed.

The transfer in of an adult male (ozM2) had a significant effect on 3 of 4 females. F2OZ showed increased mean cortisol concentrations in the 120-day time period after his transfer; whereas F3OZ and F4OZ showed decreased concentrations (Table 6). The transfers in of a juvenile female (F2OZ), adult female (F10NZ), and a group of adult females had no significant effects on cortisol concentrations in herdmates (Table 6).

The transfer out of a sub-adult male (nzM1, at age 14) had a significant effect on 2 of 4 females. F5NZ (mother) and F9NZ both showed increased mean cortisol concentration in the 30-day time period following his transfer (Table 6). F5NZ returned to pre-event concentrations after 15 days; F9NZ returned to pre-event concentrations after 30 days (Fig 5A). The transfer out of an adult female (ozF3) had no significant effect on cortisol concentrations in OZ herdmates (Table 6).

#### Transfers of self

Six females in the study were transferred, three of which were modelled. One female did not have sufficient cortisol data to model the transfer, and two females were not modelled because they did not exhibit a period of normal cycling during the study. Transfer of self was a significant predictor of mean cortisol concentration in all three modelled individuals, although responses differed (Table 7).

Females F5NZ and F8NZ showed higher mean cortisol concentrations post-transfer; whereas F9NZ showed lower concentrations (Table 7). F5NZ exhibited increased cortisol only in the first 15 days before returning to pre-transfer concentrations (Fig 5B), while F8NZ exhibited increased cortisol for 30-days (Fig 5C). By contrast, cortisol was decreased in F9NZ through at least 45 days (Fig 5D).

#### Health decline

Four females were humanely euthanized during the study; two had sufficient cortisol data to be modelled. The period of health decline leading to euthanasia was a significant predictor of mean cortisol concentration in one female (F6NZ) but not the other (F4OZ) (Table 8). F6NZ showed significantly higher mean cortisol concentration in the final 30 days of life, with a sharp increase in the week prior to euthanasia.

## Discussion

This study confirmed the presence of intrinsic cortisol patterns associated with reproductive state, ovarian cycle phase, and age in female Asian elephants, similar to that shown in other species and in previous studies of elephants [17, 37, 40, 41, 72–77]. Results further highlight the importance of accounting for these patterns when evaluating the impact of environmental factors on adrenal GC activity. Concentrations of serum and urinary cortisol covaried more consistently with physiological changes (changes in reproductive state, ovarian cycle phase, and age) than with social changes (births, deaths, and transfers), with no differences observed between the two facilities. Interestingly, effects of transfers on cortisol were related to movement of males only, suggesting the influence of bulls in a herd should be explored further. Differing responses to social changes reinforce the notion that welfare should be assessed on an individual basis, and should consider variability in GCs within individuals in addition to average concentrations.

### Reproduction and adrenal GC activity

Reproductive state (prepubertal, normal cycling, pregnant, lactational anestrus, irregular cycling, contracepted, acyclic) was a significant predictor of mean serum and urinary cortisol concentrations in 80% (4/5) of the females that changed reproductive states during the study, but the pattern varied among individuals.

#### Puberty

In the two females that reached puberty during the study, mean cortisol was lower in prepuberty compared to normal cycling for one female (zoo-born) and higher for the other (wild-born). Variability (CV) was lower in prepuberty for both females. The differences between these two females may be due in part to social factors. The zoo-born female was born into a herd and had the social support of adult female elephants from birth; whereas the prepubertal timeframe for the wild-born elephant coincided with assimilation into a herd for the first time since she was orphaned.

#### Ovarian cycle

Ovarian cycle phase was a significant predictor of mean serum and urinary cortisol concentrations in all elephants combined and in 44% (4/9) of the individual females. In general, higher mean cortisol concentration were observed in the follicular compared to the luteal phase. Although as a group, both parous/multiparous and nulliparous females exhibited this pattern, the interaction of parity and cycle phase was statistically significant, with cycle phase having a larger effect on cortisol in parous/multiparous females compared to nulliparous females, and a greater percentage of parous females exhibited higher mean values during the follicular phase. These findings confirmed cyclic patterns of serum cortisol in parous Asian elephants reported by Fanson *et al*. [37], where concentrations increased in the first half of the follicular phase, peaked around ovulation, then declined quickly and remained low throughout the luteal phase. By contrast, in nulliparous females who failed to conceive despite repeated mating or artificial insemination attempts, the cortisol pattern across the ovarian cycle was not as clear [37], a finding similar to ours. Thus, changes in GCs across the estrous cycle may promote normal ovarian function, including ovulation and formation of the corpus luteum (reviewed in Tetsuka [78]). Suppressive or deleterious effects of elevated cortisol have been a focus in studies of reproductive dysfunction in elephants [68, 79, 80]; the potential permissive or stimulatory effects of cortisol have yet to be fully explored in this species. Increase in GCs prior to or around ovulation have been found in well-studied model species [72] and in a growing number of wildlife and domesticated species (e.g., giant panda (*Ailuropoda melanoleuca*) [73], musk shrew (*Suncus murinus*) [75]; sheep [74]). In rats, the peak in GC concentration at proestrus is due to a change in the amplitude of the circadian rhythm rather than an overall upregulation of adrenal GC activity. Specifically, the morning peak in GCs is highest in proestrus and lowest immediately following ovulation, but nadir values remain the same [72, 81]. These findings imply that short-term increases in GCs may have stimulatory effects on reproductive function, and thus the pattern of GC secretion may be more biologically relevant than mean values. Monitoring the circadian variation in GC concentrations surrounding reproductive states of both sexes may help elucidate the interaction between the hypothalamic–pituitary–gonadal (HPG) and HPA axes in elephants, and with long-term monitoring how these circadian patterns change with age and the implication of those changes.

Variability (CV) in cortisol also differed across the ovarian cycle, and was significantly higher in the luteal compared to the follicular phase for all elephants combined, although only those females that showed a difference in mean cortisol between cycle phases also showed a difference in variability and the pattern varied across individuals.

#### Pregnancy

Full pregnancies were documented in two females; one exhibited a higher mean and CV in cortisol concentrations during pregnancy compared to cycling, while the other showed no significant differences between pregnant and cycling states. In other mammalian species, GC concentrations are elevated during gestation and around parturition [4, 82]. Similar gestational increases in cortisol have been found in elephants, but not consistently, whereas increases around parturition have been widely reported. Gobush *et al*. [25] found that fecal GC metabolites (fGCMs) increased with stage of gestation in free-ranging African elephants, while in Asian elephants, overall cortisol concentrations did not appear to be significantly altered during gestation, but increased sharply peripartum [40, 41, 83]. In examination of daily serum samples at the end of gestation, Meyer *et al*. [83] observed additional surges in cortisol between 8 and 11 days prior to parturition, which was supported by Kaewmanee *et al*. [84] who reported cortisol increases before parturition, 5–10 days in African elephants and 30–40 days in Asian elephants. Together, these findings imply that cortisol plays a role in gestation and parturition in elephants as in other species. Edwards and Boonstra [82] found that in most taxa, maternal free GCs increase in late gestation by means of species-specific strategies involving increased total GC secretion, decreased corticosteroid-binding globulin, or maturation and activation of fetal adrenals, all of which lead to increased exposure of the fetus to GCs essential for development. The cortisol surges observed at the end of gestation in elephants could be part of an endocrine cascade that facilitates parturition similar to that described for other mammalian species, whereby parturition is triggered by activation of the fetal HPA axis and an increase in fetal cortisol secretion (reviewed in Meyer *et al*. [83]).

#### Lactational anestrus

In the two females that gave birth during the study, mean cortisol was highest during lactational anestrus compared to all other reproductive states, which provides evidence that milk production and/or rearing a calf is physiologically and/or physically taxing. From a metabolic perspective, pregnancy and lactation are metabolically demanding due to the increased requirement for nutrients such as glucose [85, 86]. The primary role of GCs at basal concentrations is energy regulation [12], so with increased energy demands one would expect increased GC production during lactational anestrus. Parental care also can be associated with increased “stress”, albeit generally positive in nature. Pokharel et al. [77] found that fecal GC metabolites among free-ranging adult female Asian elephants were positively correlated with the number of suckling calves and lactating females in a herd, attributing elevated CGs to the stressor of predation threats to suckling calves and the metabolic and nutritional demands of lactation. Interestingly, the one female who gave birth to two calves during the study had a lower mean cortisol concentration and CV during gestation and lactational anestrus with the second calf (a male) as compared to the first (a female), which may indicate that gestation and rearing gets easier with experience. Alternatively, sex of the calf may have influenced cortisol secretion and so warrants further investigation.

### Demographic factors and adrenal GC activity

#### Age effects across females

Age was a significant predictor of serum and urinary mean cortisol concentrations in all elephants combined, with concentrations being lowest in the 0-10-year age category, higher in the age categories spanning 11–60 years with 41–60 years being highest, then lower in the oldest age category (>61 years of age). The effect of age on cortisol concentrations was confounded with reproductive state, however, so both factors should be considered when examining extrinsic effects on adrenal GC activity. Although these variables were confounded, the effect of age across all reproductive states reflects what we might see in a group of elephants with an age structure that includes females undergoing puberty or senescence, adult females that are parous and nulliparous, and females that cycle normally.

In females combined during normal cycling, mean cortisol concentration increased overall with age, and also across age categories with the lowest concentrations in the 0-10-year age category, which then increased in the 11–20 and 21–30 categories, then remained fairly steady across the 21–60 age categories. Previous studies have shown that serum cortisol also increases with age across Asian elephant bulls [38, 39]. The difference in covariance with age across all reproductive states versus limited to normal cycling may in part be due to the exclusion of the acyclic older female, F7NZ, who was the only individual that showed a negative correlation between age and cortisol concentration; and exclusion of pregnancies and lactational anestrus at ages 11–20 years.

#### Aging effects within individuals

In individual females during normal cycling, mean cortisol increased with age in 71% (5/7) of the females, decreased in the oldest female, and showed no significant change in one female. The association between cortisol and age is complex, as highlighted by dissimilar patterns in the oldest females. The female that cycled normally until she died at age 51 showed an overall increase in cortisol with age; whereas the female who cycled normally until she was contracepted at age 59 showed an overall decrease with age. She did show an increase in her last four years at ages 67–71 while was acyclic, so this increase is not related to any change in cyclicity but likely to other factors such as health issues with advancing age.

Individuals showed different patterns in the relationship between cortisol and cycle phase with age. With exception of one female, the interaction between age and cycle phase was not significant, indicating concentrations overall increased (or decreased) equally in both the follicular and luteal phases over time. However, visual inspection showed a trend towards higher rates of change in the follicular phase in the younger females versus higher rates in the luteal phase in the older females. The youngest female initially showed higher cortisol concentrations during the luteal phase, but the higher rate of increase in the follicular phase resulted in a switch about two years after her first estrous cycle such that concentrations became higher during the follicular phase, similar to the other females. Given the role of GCs in follicular development/maturation and ovulation, it stands to reason that the relationship between cortisol and ovarian activity would become stronger in the early post-pubertal years. Further investigation into the relationship between cortisol and ovarian activity over time may help elucidate any role cortisol plays in follicular development, and how this may be associated with reproductive potential.

#### Origin of birth

Surprisingly, mean cortisol concentrations did not differ between wild-born (wild caught or orphaned) and zoo-born females. Prado-Oviedo *et al*. [87] found that wild-born elephants imported to North American zoos were on average 20 years older than captive-born elephants. Consistent with those demographics, origin was confounded with age in this study as wild-born females were in the higher age categories. The one exception was the wild-born Bornean elephant (age ~9–15 years) who exhibited mean cortisol concentrations that were approximately double that of all other females, which may be attributed to challenges in her early life experience. In the wild, female elephants generally associate with their natal herd throughout their lives, so separation of mother and offspring represents a challenging life event. This female was found injured as an orphan, blinded in one eye by an apparent gunshot wound. She formed a social bond with her caretaker and had encounters with wild elephants, but it is unknown whether she had opportunities to form social bonds with other elephants before she was imported at ~6 years of age. Four of the other wild-born females in this study lived together in the same orphanage in Sri Lanka, so at least had the opportunity to interact with other elephants. In other species, cortisol secretion later in life can be influenced by early life trauma [88] or early social experience on development of the stress response [89, 90]. Estrous cycle characteristics for the Bornean female also were different; her estrous cycles were significantly longer in duration, and progesterone peaks were higher [55]. It is also plausible there are inherent genetic differences since it has been shown Bornean elephants are a genetically distinct population [91, 92], however there are no published hormone data on other Bornean females for comparison.

### Life events and adrenal GC activity

Social life events (birth to a herdmate, death of a herdmate, transfer in or out of a herdmate or self) were significant predictors of mean serum and urinary cortisol concentration in 89% (8/9) of the females and in 39% (15/38) of the events experienced by females collectively. In all but four instances, cortisol returned to pre-event concentrations within 45 days, indicating an adaptive physiological response or resilience, whereby GCs concentrations were temporarily elevated then subsequently returned to individual baseline concentrations. In general, the adrenal GC responses observed in this study were short-lived and there was no evidence of hyper- or hypo-cortisol production in any of these females.

#### Births to herdmates

Birth to a herdmate was not a significant predictor of mean cortisol concentration in that neither of the females who experienced this event type showed any difference in cortisol post-birth. Although results did not provide evidence of a change in adrenal GC activity in response to the birth in the timeframe measured, birth within an elephant herd is generally considered a positive stimulus. Calves also have a beneficial impact on a herd, stimulating play behavior and affiliative interactions [93]. Wild elephant herds typically include calves and juveniles, and there is evidence that allomothering strengthens social bonds and improves a female’s ability to successfully mother her own calves in the future [22, 94], and as such the addition of calves through successful breeding promotes normal behavior for this species in addition to giving females the opportunity to reproduce.

#### Deaths of herdmates

Death of a herdmate was a significant predictor of mean cortisol concentration in 47% (7/15) of the death events experienced by seven females collectively. The response to the same death varied among individuals, and 4 of 5 females who experienced multiple deaths exhibited different responses to the deaths of different herdmates.

Observations of zoo and wild elephants exhibiting signs of distress or showing empathetic and helping behaviors towards dying and deceased conspecifics [23, 24] suggest that death, although a natural process, can be an emotionally challenging experience. Whether a death yields an adrenal GC response likely depends on the relationship to the individual, the strength of social bonds, and social support from conspecifics or human caretakers.

In the death of a female calf (1.4 years of age), both the mother and a herdmate showed significant decreases in cortisol in the short-term. The calf’s death to elephant endotheliotropic herpesviruses was sudden, and there were no obvious signs in the days leading up to her death. Although the calf was still nursing, the mother had resumed cycling and showed no change in reproductive state after the calf’s death. There were no obvious signs of depression based on keeper records in the days following the death. One possibility is that rearing of a calf, for both mother and allomother, is associated with an increase in “stressful” stimuli, either in terms of greater physiological (e.g., lactation) or physical and psychological (e.g., calf rearing) demands, so a sudden loss of that responsibility may lead to reduced cortisol. This plausible explanation is supported by our findings that cortisol is highest during lactational anestrus. Although it is possible that lactation is a physiological stressor, the mother exhibited no prolonged elevations or depressions in cortisol concentrations during lactation, so it is unlikely the decreased cortisol after the death represented adrenal exhaustion.

#### Transfers of herdmates

Transfer of a herdmate was a significant predictor of mean cortisol concentration in 29% (5/17) of the events experienced by seven females collectively, and all were in response to transfers of males; there were no significant effects in response to transfers of female herdmates.

The lack of adrenal GC response to transfers in of adult females in this study was surprising. Disrupting stable social groups by adding or removing individuals has been shown to cause social instability and increased aggression in some species [95, 96], resulting in elevated GC production [97]. In elephants, introductions of new females have resulted in increased GCs and behavioral changes in both residents and transfers [30, 31]. It is possible the quarantine and acclimation period after transfer was too long to register a response, and measurement surrounding the social introduction phase would have yielded a stronger response.

With the transfer out of a sub-adult male, both his mother and an unrelated female showed an increase in mean cortisol. A transfer out of a herdmate constitutes a sudden change that is not predictable, with no signals of impending departure (as perceived by resident animals) such as illness or changes in behavior–a change that is not part of the natural history for this species although it may be part of an individual’s life history. In this case, the male transferred had reached puberty and would have begun to leave his natal group in the wild, and he was no longer socially integrated with the females. Although the departing individuals may no longer be socially integrated in the herd in daily life, these findings suggest that management practices should consider ways to prepare and acclimatize resident elephants after a herd mate is transferred out.

#### Transfer of self

Transfer of self was a significant predictor of mean cortisol concentration in the three individuals with sufficient data to model, with two females showing higher, and one showing lower concentrations post-transfer. All involved transfers to NZP. Female F5NZ showed an increase only in the first 15 days post-transfer, indicating she acclimated quickly. Her transfer was a short distance and she was returning to her home facility after a year-long breeding loan, and into a herd in which she had existing social bonds, so it is not surprising she adjusted so quickly. The other two females were a mother and daughter that transferred together with one other herdmate from the same facility. The transfer was a longer distance requiring multiple days of travel. The daughter, F8NZ, acclimated quickly showing an increased cortisol concentration only in the first 30 days. The mother, F9NZ, took longer to acclimate, showing decreased cortisol concentration for at least 45 days post transfer.

Because of the quarantine time requirement (c.a. 30 days), any change in adrenal GC response for a transfer of self was associated with transport and acclimation rather than social introductions to resident elephants. Transportation has been recognized as a stressor in domestic and wild animals [27, 28], so it was not surprising that short-term increases were observed. Quick acclimation has been reported in working African elephants after translocation to a new reserve [26]. Response to transportation may differ with factors including prior crate training, travel distance, and physical condition. Furthermore, prior translocations and social relationships and interactions might have modified the stress response [13], perhaps explaining why two elephants showed an increase in cortisol, while one showed a decrease post-transfer.

#### Health decline

Four females were humanely euthanized during the study. The health decline leading to euthanasia was a significant predictor of mean cortisol concentration in one of two females with sufficient data to model. One female showed significantly higher mean cortisol concentration in the final 30 days of her life compared to the previous 30 days, with a sharp increase in the last week prior to euthanasia. By contrast, the other female showed no increases prior to euthanasia. It is possible that difference in responses could be attributed to the nature and intensity of the health decline and the point at which the euthanasia decision was made. Both suffered from chronic foot and joint problems that were exacerbated over time and became less responsive to anti-inflammatory treatments. In the period leading up to euthanasia, F6NZ increasingly leaned on objects/walls to relieve pain, whereas F4OZ’s decline was more gradual and the decision to euthanize may have been made at a different point in decline.

### Individuality in adrenal GC response

Adrenal cortisol responsiveness clearly varied across individuals, with each being exposed to two to seven social life events. In six females with over 10 years of cortisol data, the percentage of significant responses to social life events ranged from 0 to 50%. These extremes were observed in two females at NZP, each of whom experienced six social events with five events that were the same. Female F5NZ was the most responsive to deaths and transfers, whereas the oldest female in the study, F7NZ, did not show a significant response to any of the six events she experienced. Social relationships likely played a role in that the death and transfer out of a herdmate that yielded an effect were both offspring to F5NZ.

Individual variation in GC response to exogenous stimuli is ubiquitous. Cockrem [98] presents studies that show mean plasma cortisol concentrations increase in response to capture, restraint, or confinement in all vertebrate groups (fish, amphibians, reptiles, birds, mammals), but within each group there is extensive individual variation in response to the same stressor. In mammals, endogenous factors that lead to differences in GC responses include genetic variation and maternal influences before and after birth (reviewed in Cockrem [98] and Palme [6]).

Individuals have different personalities and temperaments or coping styles, and individual variation in GC response has been linked to coping styles described as proactive/bold and reactive/shy in laboratory and farm animals [99, 100]. Fanson *et al*. [27] found that individual patterns of adrenocortical activity in response to long-distance relocation of female Asian elephants were at least partially explained by differences in elephant behavioral traits or temperament. Grand et al. [101] found differences in cortisol response patterns to be correlated with fearful, effective, sociable, and aggressive personality components in a small group of captive African elephants. Temperament may have played a role in F7NZ’s apparent lack of adrenal GC response to numerous life events. Caretakers described her as “a very wise elephant who socializes well with both elephants and people. She is careful and does not like change, but copes with it well by letting others experience things first, and then she will follow”.

Aspects of social environment (group structure, degree of social integration or isolation, dominance rank) are important factors in how an individual or group responds to social events, and these factors have been shown to exert influence on the adrenal GC response in many social species [89, 90, 102]. Social support may offer protective or buffering effects against stressful challenges [103]. Increasing evidence suggests that affiliative behaviors in some animals can provide a buffer against stress and have a positive impact on measures of health and well-being [104]. The capacity for reassurance and empathy towards conspecifics in distress has been shown in Asian elephants [105]. It is plausible in this study that lack of physical access to herdmates giving birth or dying could have influenced adrenal GC response by limiting affiliative tactile interactions; however, vocalizations and other affiliative behaviors may provide reassurance, and the remainder of the herd generally had physical access to each other during these events. Degree of familiarity and stability of social bonds are factors influencing social support, but are not necessarily required [103]. Differences between African and Asian elephants in social aspects of their natural history may shed light on the observed responses, or lack thereof. African elephants form complex, multi-tiered social groups that are important to survival, whereas Asian herds are smaller and bonds are more fluid [106], thus the response to social changes in Asian elephants may not be as pronounced as in African elephants [107]. The influence of dominance rank on adrenal GC response was not measured because dominance hierarchies among females in this study were weak and non-linear as seen in free-ranging Asian elephants [108], the rank of some individuals changed over time, and there were periods when dominance hierarchy was in flux, e.g., after the death or transfer of a dominant female.

Finally, individual differences in adrenal GC response to social change may be attributed in part to the human-animal relationships that allow each to make predictions about the other, and human-animal bonds that have been demonstrated in both stockmanship and zoo keeping [109, 110]. Zoo elephants are highly managed, and therefore caretakers are an important facet of elephants’ daily lives. The manner in which caretakers look after their elephants and how elephants respond in preparation for or following potentially stressful events may influence an individual elephant’s response to those events.

### Conclusion

This retrospective study furthers our understanding of the role of cortisol in ovarian function and emphasizes the importance of taking intrinsic patterns of cortisol secretion into account when assessing adrenal GC responses to external stimuli. Results also highlight the importance of routine blood sampling for long-term monitoring of reproductive and adrenal activity. While this study focused on cortisol as an indicator of adrenal function, measures of other adrenal hormones, like dehydroepiandrosterone (DHEA) and dopamine, in females across different reproductive states could shed light on additional intrinsic patterns and possible covariance with cortisol in relation to reproduction. The role of dopamine also should be explored due to its inhibitory control of prolactin and known relationship with ovarian dysfunction in African elephants [111]. Furthermore, age-related changes in DHEA and adrenal hormones could provide insight into the relationship between adrenal activity and metabolic changes, and aid in the care of geriatric elephants.

Because social life events can disrupt stable social groups and social bonds, it was surprising that less than 40% of social life events altered GC activity. Although there are differences in the social environment of *in-situ* and *ex-situ* elephant populations, zoo-housed females form strong social bonds [112], the individuals in this study experienced strong bonds and long-term companionship, some but not all were genetically related, and there was group stability across multiple years, thus reflecting the more fluid society with smaller group sizes found in Asian elephant herds *in-situ* [108, 113]. The low rate of change in GC activity reflects only one measure of response to social change. Thus, an integrated approach using both behavioral and physiological measures is necessary to fully understand how animals perceive and interact with changes or challenges in their environment [114, 115]. Further investigations could include examining the underlying mechanisms through which social behavior, including both animal-animal and human-animal interactions, may provide a buffer against such stressors. For example, measures of oxytocin, a neuropeptide known for promoting social behavior through which positive social interactions suppress the HPA axis (reviewed in DeVries *et al*. [104]), could clarify the role of prosocial behavior and social support in how individual elephants respond to stressors.

Indivduality in adrenal GC response to social life events provides evidence that life history, social relationships, temperament, and social support are important factors influencing the impact of perceived stressors. The observed response of females to transfers in and out of males reinforces the important role of males in the herd even if they are not always socially integrated. Pinto et al [116] concludes that even temporary integration of a male elephant into a female group in captivity has positive impacts in females, and that further studies should be done on the influence of males on welfare of females in captivity. Finally, an understanding of the impact of life events and resiliency to perceived stressors would be beneficial in determining the welfare needs of individuals and groups.

## Supporting information

S1 Table Serum cortisol concentration (ng/ml) for females across all reproductive states. Individual, birth date and origin, housing facility, age range during study, parity, number of samples measured for serum cortisol, and concentration median, range, mean, and standard deviation (SD).
(DOCX)

S2 Table Urinary cortisol concentration (ng/mg Cr) for females across all reproductive states. Individual, birth date and origin, housing facility, age range during study, parity, number of samples measured for urinary cortisol, and concentration median, range, mean, and standard deviation (SD).
(DOCX)

S3 Table Serum cortisol concentration (ng/ml) for females during periods of normal ovarian cyclicity. Individual, birth date and origin, housing facility, age range during study, parity, number of samples measured for serum cortisol, and concentration median, range, mean, and standard deviation (SD).
(DOCX)

S4 Table Urinary cortisol (ng/mg Cr) for females during periods of normal ovarian cyclicity. Individual, birth date and origin, housing facility, age range during study, parity, number of samples measured for urinary cortisol, and concentration median, range, mean, and standard deviation (SD).
(DOCX)

S5 Table Coefficient of variation (CV) in cortisol concentration for reproductive states. Individual, CV percentage for reproductive state, Brown-Forsythe Test statistic, and p-value for comparisons.
(DOCX)

S6 Table Coefficient of variation (CV) in cortisol concentration for ovarian cyclicity. Individual, sample, CVs for ovarian cycle phases, Brown-Forsythe Test statistic, and p-value.
(DOCX)

S1 Fig Ovarian cycle phase as a predictor of adrenal GC activity in individuals. Predictions from GLMMs for mean cortisol concentrations in the luteal phase and follicular phase of the ovarian cycle for individuals (error bars represent standard error of the prediction). Letters denote significant differences in hormone concentration between cycle phases. (A) F1OZ. (B) F7NZ. (C) F5NZ: serum cortisol. (D) F5NZ: urinary cortisol. (E) F8NZ.
(TIF)

S2 Fig Death of a herdmate as a predictor of adrenal GC activity. Predictions from GLMMs for mean cortisol concentration in the 30 days prior to death and the 30 days post-death (error bars represent standard error of the prediction). Letters denote a significant difference in hormone concentration between pre- and post-death. (A) Death 1, F2OZ response: post > pre, comparing 30 days pre-post death. (B) Death 1, F3OZ response: post > pre, comparing 30 days pre-post death. (C) Death 4, F4OZ response: post > pre, comparing 30 days pre-post death.
(TIF)

S3 Fig Transfer in of a herdmate a predictor of adrenal GC activity. Predictions from GLMMs for mean cortisol concentration in the 120 days prior to transfer and 120 days post-transfer in (error bars represent standard error of the prediction). Letters denote a significant difference in hormone concentration between pre- and post-transfer. (A) Transfer 2, F2OZ response: post > pre, comparing 120 days pre-post transfer. (B) Transfer 2, F3OZ response: post < pre, comparing 120 days pre-post transfer. (C) Transfer 2, F4OZ response: post < pre, comparing 120 days pre-post transfer.
(TIF)

S4 Fig Transfer out of a herdmate as a predictor of adrenal GC activity. Predictions from GLMMs for mean cortisol concentrations in the 30 days prior to transfer and 30 days post-transfer (error bars represent standard error of the prediction). Letters denote a significant difference in hormone concentration between pre- and post-transfer. (A) Transfer 6, F5NZ response: post > pre, comparing 30 days pre-post transfer. (B) Transfer 6, F9NZ response: post > pre, comparing 30 days pre-post transfer.
(TIF)

S5 Fig Transfer in of self as a predictor of adrenal GC activity. Predictions from GLMMs for mean cortisol concentrations in the 30 days prior to transfer and 30 days post-transfer (error bars represent standard error of the prediction). Letters denote a significant difference in hormone concentration between pre**-** and post-transfer. (A) Transfer of self, F8NZ: post > pre, comparing 30 days pre-post transfer. (B) Transfer of self, F9NZ: post > pre, comparing 30 days pre-post transfer.
(TIF)
