# Supplementary material for: Effects of physiological changes and social life events on adrenal glucocorticoid activity in female zoo-housed Asian elephants (Elephas maximus)
Source: PLoS One. 2020 Nov 6;15(11):e0241910. doi: 10.1371/journal.pone.0241910 (PMC7647113; doi:10.1371/journal.pone.0241910)
Supplement: S2 Table — Individual, birth date and origin, housing facility, age range during study, parity, number of samples measured for urinary cortisol, and concentration median, range, mean, and standard deviation (SD). (DOCX) [file pone.0241910.s002.docx]

S2 Table: Urinary cortisol concentration (ng/mg Cr) for females across all reproductive states. Individual, birth date and origin, housing facility, age range during study, parity, number of samples measured for urinary cortisol, and concentration median, range, mean, and standard deviation (SD).

|  |  |  |  | **Urinary cortisol (ng/mg Cr) – All repro states** | | | |
| --- | --- | --- | --- | --- | --- | --- | --- |
| **Individual** | **Origin / Birthdate / Housing facility** | **Age range (years)** | **Parity** | **N** | **Median (range)** | **Mean (SD)** | **CV%** |
| F5NZ | Wild (Sri Lanka)  ~1976 National Zoo | ~39–43 | Multiparous | 215 | 202.29  (15.88 – 957.76) | 228.49 (132.91) | 39.4 |
| F7NZ | Wild (India)  ~1948 National Zoo | ~68–71 | Nulliparous | 275 | 240.62  (20.43 – 1377.78 | 269.77 (161.93) | 60.0 |
| F8NZ | Zoo-born  7/14/90 National Zoo | 24–28 | Multiparous | 166 | 197.23  (0.08 – 1409.60) | 271.73 (242.19) | 89.1 |
| F9NZ | Wild (Sri Lanka)  ~Sep 1975 National Zoo | ~39–43 | Multiparous | 174 | 171.72  (33.13 – 952.27) | 200.45 (129.97) | 64.8 |
| F10NZ | Wild (Sri Lanka)  ~1975 National Zoo | ~38–42 | Nulliparous | 233 | 185.74  (42.78 – 1000.00) | 241.42 (176.23) | 73.0 |
| F11NZ | Wild (Sri Lanka)  ~ Aug1975 National Zoo | ~38–43 | Nulliparous | 242 | 430.62  (54.00 – 3830.12) | 568.86 (519.32) | 91.3 |
| All Elephants |  | 24 to ~71 |  | 1305 | 223.08  (0.08 – 3830.12) | 304.38 (299.85) | 98.5 |
