# Supplementary material for: Effects of physiological changes and social life events on adrenal glucocorticoid activity in female zoo-housed Asian elephants (Elephas maximus)
Source: PLoS One. 2020 Nov 6;15(11):e0241910. doi: 10.1371/journal.pone.0241910 (PMC7647113; doi:10.1371/journal.pone.0241910)
Supplement: S3 Table — Individual, birth date and origin, housing facility, age range during study, parity, number of samples measured for serum cortisol, and concentration median, range, mean, and standard deviation (SD). (DOCX) [file pone.0241910.s003.docx]

S3 Table: Serum cortisol concentration (ng/ml) for females during periods of normal ovarian cyclicity. Individual, birth date and origin, housing facility, age range during study, parity, number of samples measured for serum cortisol, and concentration median, range, mean, and standard deviation (SD).

|  |  |  |  | **Cycling normally** | | | | **Luteal phase** | | **Follicular phase** | |
| --- | --- | --- | --- | --- | --- | --- | --- | --- | --- | --- | --- |
| **Individual** | **Origin / Birthdate / Housing facility** | **Age range during cycling (years)** | **Parity** | **N** | **Median  (range)** | **Mean (SD)** | **Median  (range)** | | **Mean (SD)** | **Median (range)** | **Mean (SD)** |
| F1OZ | Zoo-born 8/31/1994 Oregon Zoo | 4–14 | Parous | 181 | 10.50  (2.50 – 67.40) | 13.19 (9.71) | 9.50 (2.50 – 67.40) | | 11.41 (8.07) | 13.10 (2.50 – 51.80) | 15.39 (11.08) |
| F2OZ | Wild (Borneo) ~1993 Oregon Zoo | ~12–15 | Nulliparous | 166 | 26.30  (2.50 – 77.30) | 28.13 (16.21) | 25.20  (2.50 – 69.50) | | 28.56 (16.44) | 26.90  (2.50 – 77.30) | 27.49 (15.95) |
| F3OZ | Zoo-born 12/26/82 Oregon Zoo | 12–26 | Nulliparous | 348 | 11.80  (2.50 – 77.60) | 15.10 (12.37) | 10.40  (2.50 – 77.60) | | 14.42 (11.77) | 13.80  (2.50 – 63.40) | 15.89 (11.85) |
| F4OZ | Wild (Thailand) ~1955 Oregon Zoo | ~39–51 | Multiparous | 488 | 12.25  (2.50 – 82.80) | 16.30 (13.02) | 12.65  (2.50 – 74.35) | | 16.39 (12.61) | 12.05  (2.50 – 82.80) | 16.32 (13.03) |
| F5NZ | Wild (Sri Lanka)  ~1976 National Zoo | ~15–43 | Multiparous | 721 | 13.00 (2.50 – 96.00) | 15.97 (11.01) | 12.10  (2.50 – 79.30) | | 14.73 (10.47) | 14.90  (2.50 – 96.00) | 18.27 (11.62) |
| F6NZ | Wild (Thailand)  ~1965 National Zoo | ~27–41 | Nulliparous | 576 | 11.10  (2.50 – 74.10) | 12.77 (7.38) | 10.98  (2.50 – 74.10) | | 12.74 (7.53) | 11.20  (2.50 – 40.40) | 12.83 (7.06) |
| F7NZ | Wild (India)  ~1948 National Zoo | ~46–59 | Nulliparous | 509 | 12.78  (2.50 – 56.51) | 14.96 (8.39) | 12.13  (2.50 – 56.51) | | 14.07 (7.57) | 15.11  (2.50 – 54.27) | 17.04 (9.26) |
| All Elephants |  | 4 to ~59 |  | 2989 | 12.37 (2.50 – 96.00) | 15.64 (11.32) | 11.84  (2.50 – 79.30) | | 14.94 (10.88) | 13.70  (2.50 – 96.00) | 16.88 (11.66) |
