# Supplementary material for: Effects of physiological changes and social life events on adrenal glucocorticoid activity in female zoo-housed Asian elephants (Elephas maximus)
Source: PLoS One. 2020 Nov 6;15(11):e0241910. doi: 10.1371/journal.pone.0241910 (PMC7647113; doi:10.1371/journal.pone.0241910)
Supplement: S4 Table — Individual, birth date and origin, housing facility, age range during study, parity, number of samples measured for urinary cortisol, and concentration median, range, mean, and standard deviation (SD). (DOCX) [file pone.0241910.s004.docx]

S4 Table: Urinary cortisol (ng/mg Cr) for females during periods of normal ovarian cyclicity. Individual, birth date and origin, housing facility, age range during study, parity, number of samples measured for urinary cortisol, and concentration median, range, mean, and standard deviation (SD).

|  |  |  |  | **Cycling normally** | | | **Luteal phase** | | **Follicular phase** | |
| --- | --- | --- | --- | --- | --- | --- | --- | --- | --- | --- |
| **Individual** | **Origin / Birthdate / Housing facility** | **Age range during cycling (years)** | **Parity** | **N** | **Median  (range)** | **Mean (SD)** | **Median  (range)** | **Mean (SD)** | **Median (range)** | **Mean (SD)** |
| F5NZ | Wild (Sri Lanka)  ~1976 National Zoo | ~39–43 | Multiparous | 186 | 203.00  (15.88 – 957.76) | 233.69 (137.70) | 188.76  (15.88 – 957.76) | 213.84 (122.03) | 244.80 (59.14 – 867.62) | 271.54 (157.66) |
| F8NZ | Zoo-born  7/14/90 National Zoo | 24–28 | Multiparous | 166 | 197.23  (0.08 – 1409.60) | 271.73 (242.19) | 153.04 (0.08 – 1409.60) | 268.34 (236.18) | 261.53 (53.35 – 1000.00) | 340.11 (234.22) |
| F9NZ | Wild (Sri Lanka)  ~Sep 1975 National Zoo | ~39–43 | Multiparous | 152 | 191.25 (33.13 – 952.27) | 211.64 (133.06) | 188.36  (33.13 – 952.27) | 207.13 (136.98) | 198.17  (35.23 – 591.43) | 220.57 (125.79) |
| All Elephants |  | 24 to ~43 |  | 504 | 197.55  (0.08 – 1409.60) | 239.57 (179.22) | 186.26  (0.08 – 1409.60) | 216.22 (163.30) | 242.18  (35.23 – 1000.00) | 276.56 (181.60) |
