# Supplementary material for: Effects of physiological changes and social life events on adrenal glucocorticoid activity in female zoo-housed Asian elephants (Elephas maximus)
Source: PLoS One. 2020 Nov 6;15(11):e0241910. doi: 10.1371/journal.pone.0241910 (PMC7647113; doi:10.1371/journal.pone.0241910)
Supplement: S5 Table — Individual, CV percentage for reproductive state, Brown-Forsythe Test statistic, and p-value for comparisons. (DOCX) [file pone.0241910.s005.docx]

S5 Table: Coefficient of variation (CV) in cortisol concentration for reproductive states. Individual, CV percentage for reproductive state, Brown-Forsythe Test statistic, and p-value for comparisons

| **Individual** | **CV%** | | | | | | | **BF Test** | |
| --- | --- | --- | --- | --- | --- | --- | --- | --- | --- |
|  | **Prepubertal** | **Cycling** | **Pregnant** | **Lactation Anestrous** | **Contracepted / Acyclic** | **Irregular Cycling** |  | |  |
| F1OZ | 56.2 | 74.2 | 81.2 | 87.8 |  |  | *F**(3, 115) = 22.83, **p = <0.001** | |  |
|  | x | x |  |  |  |  | *F**(1, 99.0) = 12.15, **p = < 0.001** | |  |
|  | x |  | x |  |  |  | *F**(1, 95.0) = 20.63, **p = < 0.001** | |  |
|  | x |  |  | x |  |  | *F**(1, 51.9) = 50.68, **p = < 0.001** | |  |
|  |  | x | x |  |  |  | *F**(1, 92.8) = 6.95, **p = 0.010** | |  |
|  |  | x |  | x |  |  | *F**(1, 50.0) = 34.37, **p = < 0.001** | |  |
|  |  |  | x | x |  |  | *F**(1, 78.3) = 12.92, **p = < 0.001** | |  |
| F2OZ | 55.2 | 57.6 |  |  |  |  | *F**(1, 95.9) = 7.17, **p = 0.009** | |  |
| F5NZ  (serum) |  | 70.0 | 55.0 | 46.8 | 79.5 |  | *F**(3, 187) = 3.04, **p = <0.030** | |  |
|  |  | x | x |  |  |  | *F**(1, 251) = 1.69, p = 0.194 | |  |
|  |  | x |  | x |  |  | *F**(1, 38.3) = 7.53, **p = 0.009** | |  |
|  |  | x |  |  | x |  | *F**(1, 80.3) = 0.362, p = 0.549 | |  |
|  |  |  | x | x |  |  | *F**(1, 46.9) = 10.19, **p = 0.003** | |  |
|  |  |  | x |  | x |  | *F**(1, 102) = 0.008, p = 0.929 | |  |
|  |  |  |  | x | x |  | *F**(1,82.7x) = 6.42, **p = 0.013** | |  |
| F5NZ  (urine) |  | 59.2 |  |  | 44.9 |  | *F**(1, 48.6) = 2.79, p = 0.102 | |  |
| F7NZ |  | 55.8 |  |  | 70.5 |  | *F**(1, 574) = 0.045, p = 0.832 | |  |
| F9NZ |  | 62.9 |  |  |  | 55.5 | *F**(1, 48.4) = 23.8, **p = < 0.001** | |  |

X denotes pair-wise comparisons made in the Brown-Forsythe statist
