# Supplementary material for: Effects of physiological changes and social life events on adrenal glucocorticoid activity in female zoo-housed Asian elephants (Elephas maximus)
Source: PLoS One. 2020 Nov 6;15(11):e0241910. doi: 10.1371/journal.pone.0241910 (PMC7647113; doi:10.1371/journal.pone.0241910)
Supplement: S6 Table — Individual, sample, CVs for ovarian cycle phases, Brown-Forsythe Test statistic, and p-value. (DOCX) [file pone.0241910.s006.docx]

S6 Table: Coefficient of variation (CV) in cortisol concentration for ovarian cyclicity. Individual, sample, CVs for ovarian cycle phases, Brown-Forsythe Test statistic, and p-value.

| **Individual** | **Sample** | **CV%** | | **BF Test** |
| --- | --- | --- | --- | --- |
|  |  | **Follicular** | **Luteal** |  |
| All elephants | Serum | 69.1 | 72.8 | *F**(1, 1963) = 19.42, **p = <0.001** |
| All elephants (all parous) | Urine | 65.7 | 75.5 | *F**(1, 303) = 12.82, **p = <0.001** |
| Parous females | Serum | 69.8 | 74.5 | *F**(1, 913) = 11.45, **p = <0.001** |
| Nulliparous females | Serum | 68.3 | 71.4 | *F**(1, 1046) = 8.12, **p = 0.004** |
| F1OZ | Serum | 72.0 | 70.7 | *F*(*1, 142) = 7.31, **p = 0.008** |
| F5NZ | Serum | 63.6 | 71.1 | *F*(*1, 470) = 16.28, **p = <0.001** |
| F5NZ | Urine | 58.1 | 57.1 | *F*(*1, 104) = 6.52, **p = 0.012** |
| F7NZ | Serum | 54.3 | 53.8 | *F*(*1, 241) = 11.65, **p = 0.007** |
| F8NZ | Urine | 68.9 | 88.0 | *F*(*1, 96.8) = 7.53, **p = 0.007** |
| F3OZ | Serum | 74.6 | 89.4 | *F**(1, 324) = 1.18, p = 0.278 |
| F2OZ | Serum | 58.0 | 57.6 | *F*(*1, 145) = 0.174, p = 0.676 |
| F4OZ | Serum | 79.8 | 76.9 | *F*(*1, 268) = 0.003, p = 0.954 |
| F6NZ | Serum | 55.0 | 59.1 | *F*(*1, 355) = 0.209, p = 0.648 |
| F9NZ | Urine | 57.0 | 66.1 | *F*(*1, 108) = 0.364, p = 0.547 |
