# Supplementary figures and images for: Effects of physiological changes and social life events on adrenal glucocorticoid activity in female zoo-housed Asian elephants (Elephas maximus)
Source: PLoS One. 2020 Nov 6;15(11):e0241910. doi: 10.1371/journal.pone.0241910 (PMC7647113; doi:10.1371/journal.pone.0241910)

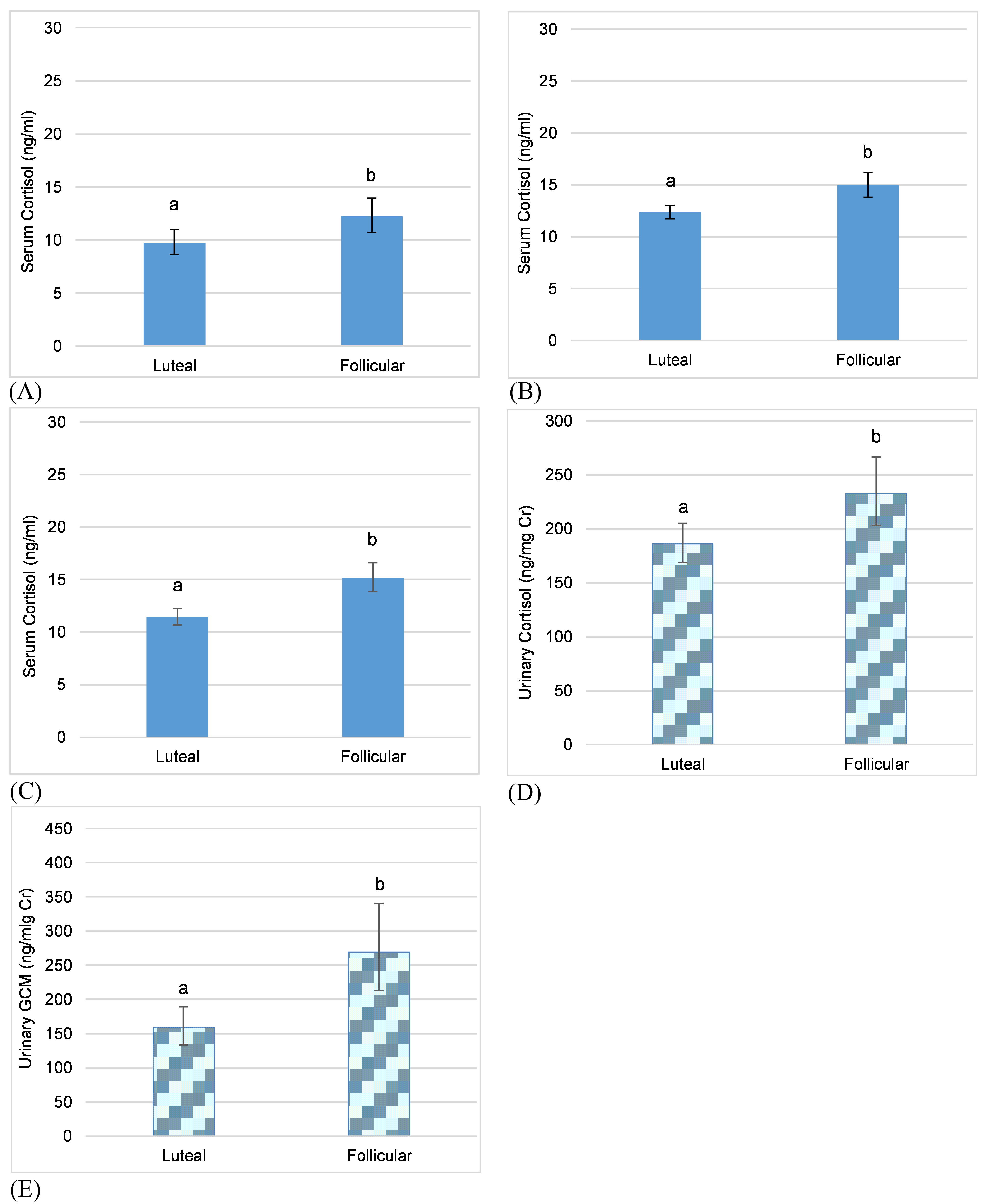

Supplement: S1 Fig — Predictions from GLMMs for mean cortisol concentrations in the luteal phase and follicular phase of the ovarian cycle for individuals (error bars represent standard error of the prediction). Letters denote significant differences in hormone concentration between cycle phases. (A) F1OZ. (B) F7NZ. (C) F5NZ: serum cortisol. (D) F5NZ: urinary cortisol. (E) F8NZ. (TIF) [file pone.0241910.s007.tif]

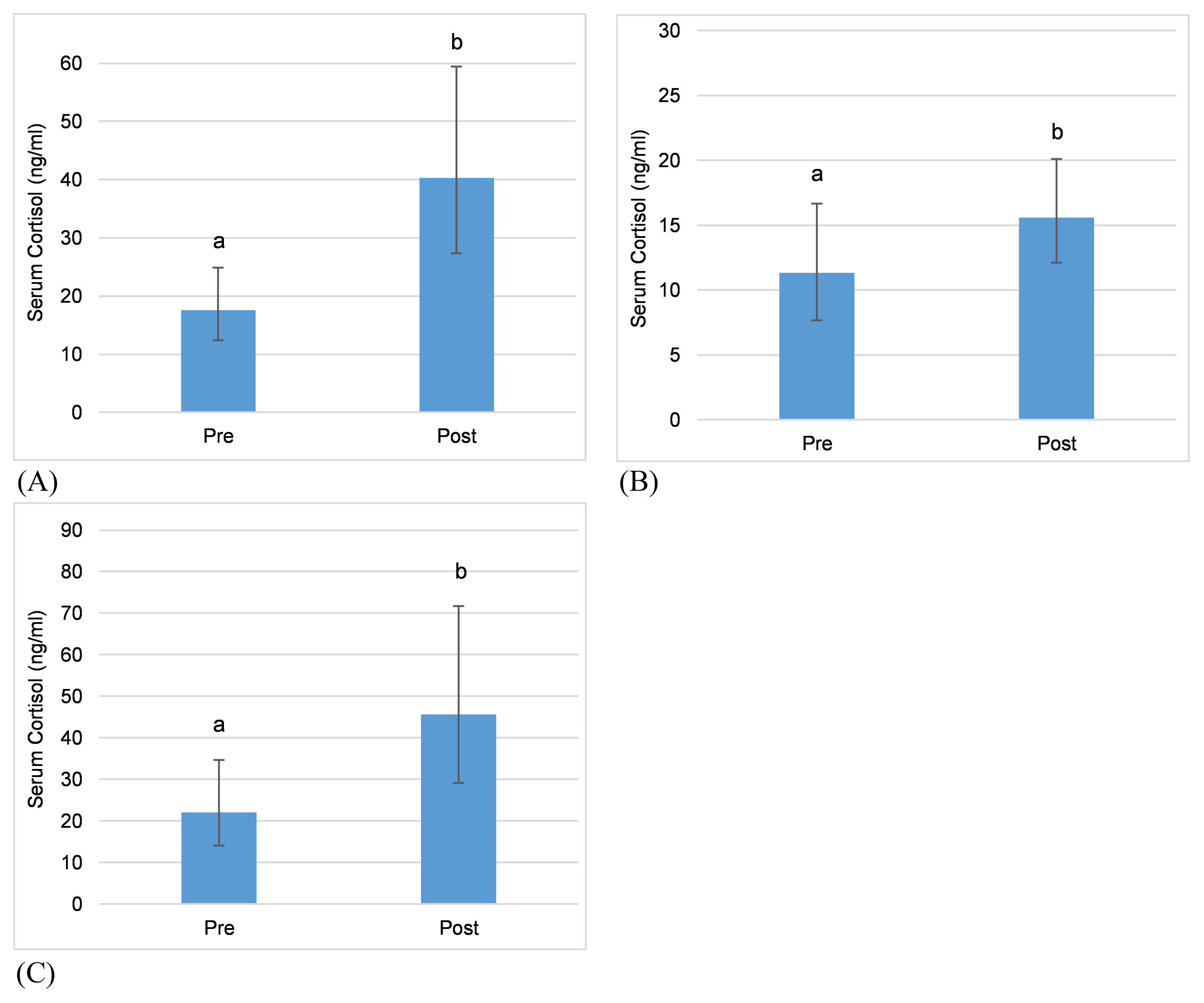

Supplement: S2 Fig — Predictions from GLMMs for mean cortisol concentration in the 30 days prior to death and the 30 days post-death (error bars represent standard error of the prediction). Letters denote a significant difference in hormone concentration between pre- and post-death. (A) Death 1, F2OZ response: post > pre, comparing 30 days pre-post death. (B) Death 1, F3OZ response: post > pre, comparing 30 days pre-post death. (C) Death 4, F4OZ response: post > pre, comparing 30 days pre-post death. (TIF) [file pone.0241910.s008.tif]

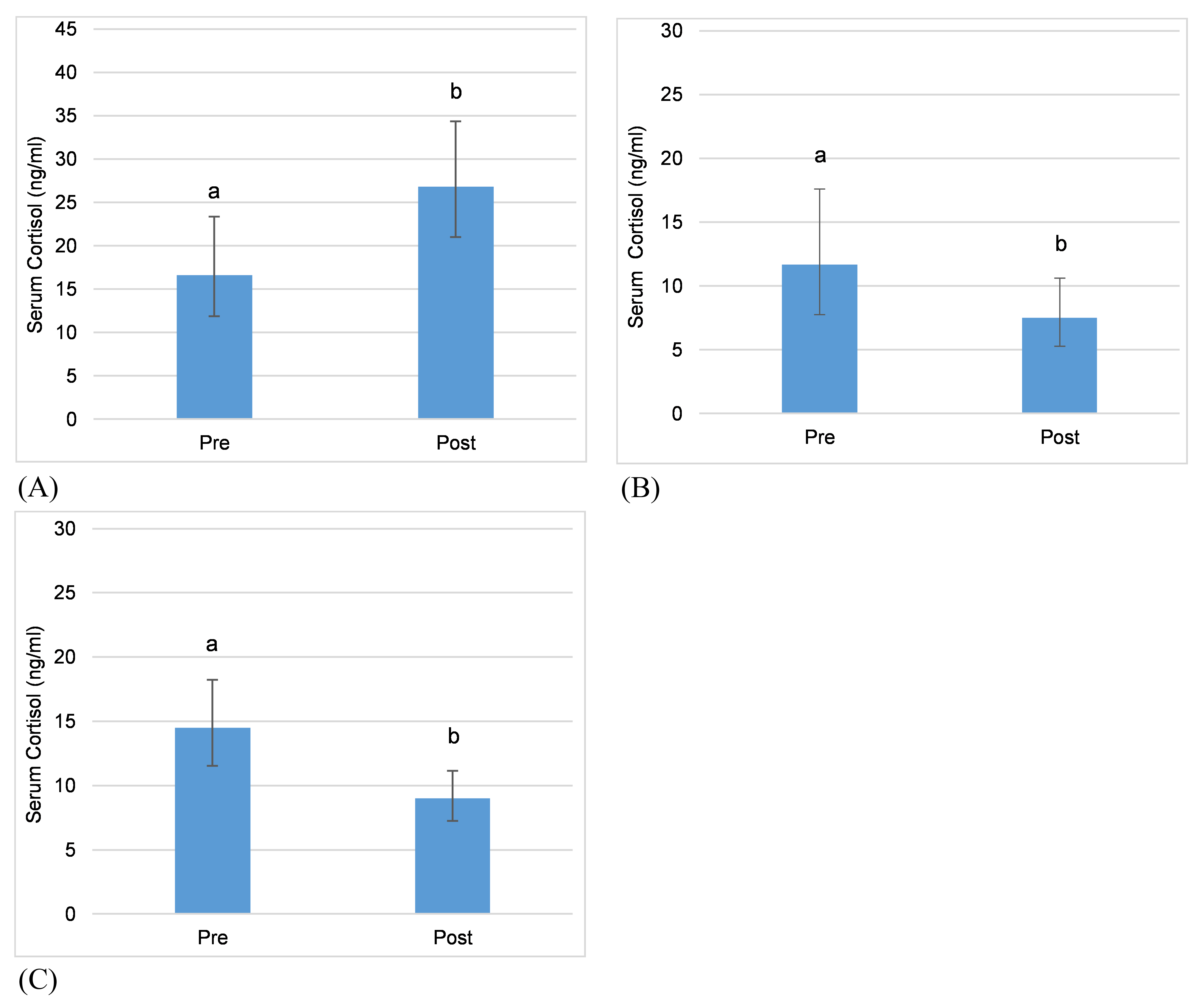

Supplement: S3 Fig — Predictions from GLMMs for mean cortisol concentration in the 120 days prior to transfer and 120 days post-transfer in (error bars represent standard error of the prediction). Letters denote a significant difference in hormone concentration between pre- and post-transfer. (A) Transfer 2, F2OZ response: post > pre, comparing 120 days pre-post transfer. (B) Transfer 2, F3OZ response: post < pre, comparing 120 days pre-post transfer. (C) Transfer 2, F4OZ response: post < pre, comparing 120 days pre-post transfer. (TIF) [file pone.0241910.s009.tif]

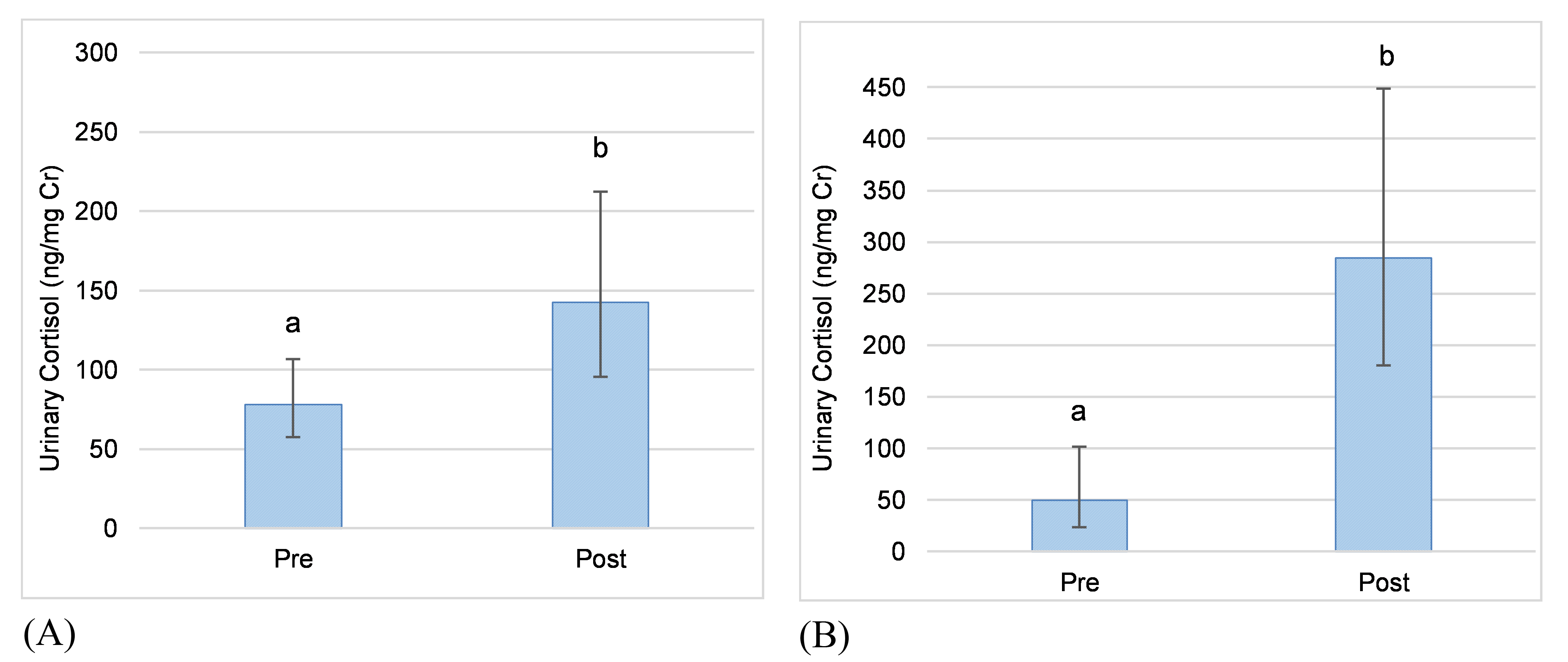

Supplement: S4 Fig — Predictions from GLMMs for mean cortisol concentrations in the 30 days prior to transfer and 30 days post-transfer (error bars represent standard error of the prediction). Letters denote a significant difference in hormone concentration between pre- and post-transfer. (A) Transfer 6, F5NZ response: post > pre, comparing 30 days pre-post transfer. (B) Transfer 6, F9NZ response: post > pre, comparing 30 days pre-post transfer. (TIF) [file pone.0241910.s010.tif]

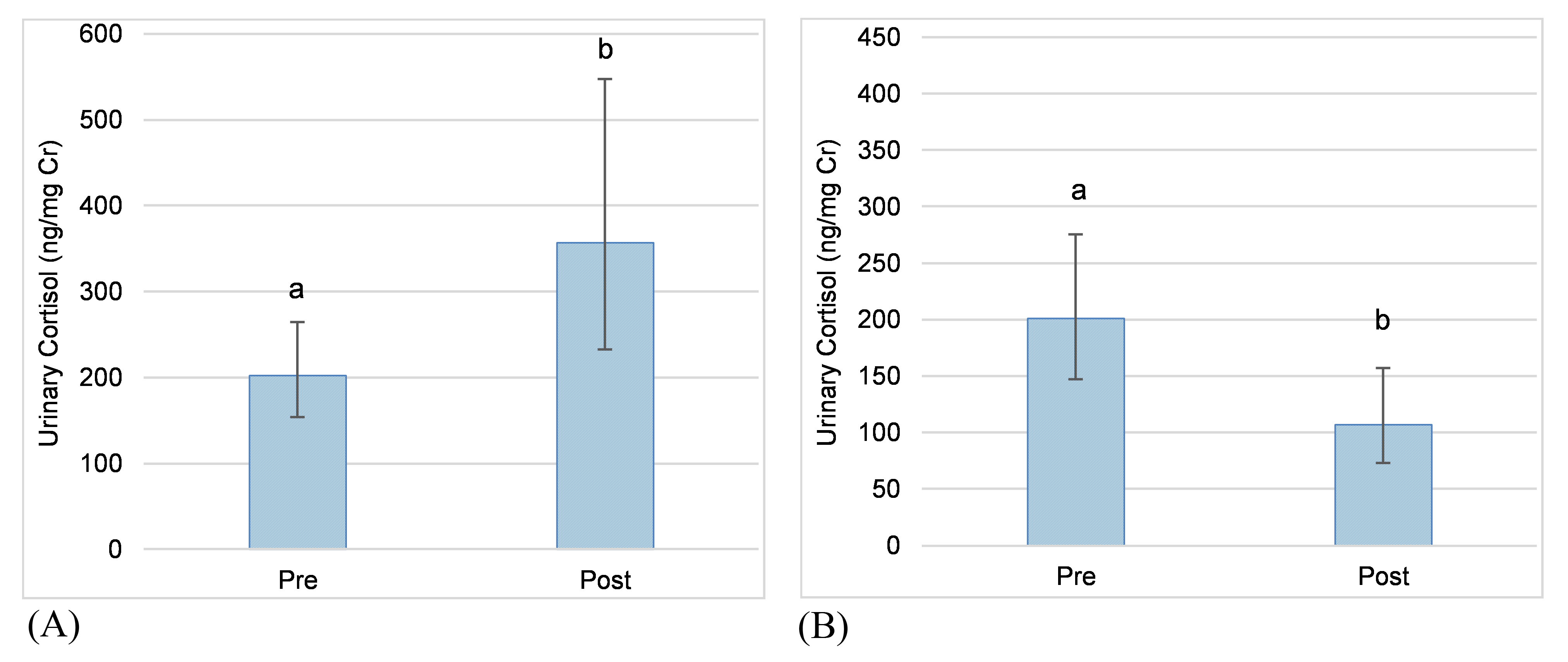

Supplement: S5 Fig — Predictions from GLMMs for mean cortisol concentrations in the 30 days prior to transfer and 30 days post-transfer (error bars represent standard error of the prediction). Letters denote a significant difference in hormone concentration between pre- and post-transfer. (A) Transfer of self, F8NZ: post > pre, comparing 30 days pre-post transfer. (B) Transfer of self, F9NZ: post > pre, comparing 30 days pre-post transfer. (TIF) [file pone.0241910.s011.tif]
